# Growth inhibition and apoptosis in cancer cells induced by polyphenolic compounds of *Acacia hydaspica*: Involvement of multiple signal transduction pathways

**DOI:** 10.1038/srep23077

**Published:** 2016-03-15

**Authors:** Tayyaba Afsar, Janeen H. Trembley, Christine E. Salomon, Suhail Razak, Muhammad Rashid Khan, Khalil Ahmed

**Affiliations:** 1 Department of Biochemistry, Faculty of Biological Sciences, Quaid-i-Azam University, Islamabad, Pakistan; 2 Cellular and Molecular Biochemistry Research Laboratory (151), Minneapolis VA Health Care System, Minneapolis, MN USA; 3 Department of Laboratory Medicine and Pathology, University of Minnesota, Minneapolis, MN USA; 4 Centre for Drug Design, University of Minnesota, Minneapolis, MN USA; 5 Department of Animal Sciences, Faculty of Biological Sciences, Quaid-i-Azam University, Islamabad, Pakistan

## Abstract

*Acacia hydaspica* R. Parker is known for its medicinal uses in multiple ailments. In this study, we performed bioassay-guided fractionation of cytotoxic compounds from *A. hydaspica* and investigated their effects on growth and signaling activity in prostate and breast cancer cell lines. Four active polyphenolic compounds were identified as 7-*O*-galloyl catechin (GC), catechin (C), methyl gallate (MG), and catechin-3-*O*-gallate (CG). The four compounds inhibited prostate cancer PC-3 cell growth in a dose-dependent manner, whereas CG and MG inhibited breast cancer MDA-MB-231 cell growth. All tested compounds inhibited cell survival and colony growth in both cell lines, and there was evidence of chromatin condensation, cell shrinkage and apoptotic bodies. Further, acridine orange, ethidium bromide, propidium iodide and DAPI staining demonstrated that cell death occurred partly via apoptosis in both PC-3 and MDA-MB-231 cells. In PC-3 cells treatment repressed the expression of anti-apoptotic molecules Bcl-2, Bcl-xL and survivin, coupled with down-regulation of signaling pathways AKT, NFκB, ERK1/2 and JAK/STAT. In MDA-MB-231 cells, treatment induced reduction of CK2α, Bcl-xL, survivin and xIAP protein expression along with suppression of NFκB, JAK/STAT and PI3K pathways. Our findings suggest that certain polyphenolic compounds derived from *A. hydaspica* may be promising chemopreventive/therapeutic candidates against cancer.

Adenocarcinomas of prostate and breast are frequently diagnosed malignancies in developed countries and are among the leading causes of cancer-related deaths in North America and Europe^1^. The prevalence of these cancers is also increasing in developing countries^2^. The development of prostate cancer (PCa) follows different stages; initially, the disease is androgen-responsive and androgen ablation is the first line of therapy, but eventually the disease progresses to an aggressive castration-resistant phenotype (i.e., unresponsive to androgens despite the presence of androgen receptor). At this stage the disease is generally unresponsive to available therapies and thus is usually fatal^3^. Breast cancer (BCa) tumors are comprised of phenotypically heterogeneous populations of cells, arising from different gene mutations occurring in luminal or basal progenitor cell population^4^. PC-3 and MDA-MB- 231 cells have been employed as models for various experimental studies, and since these cells express a variety of deregulated signaling pathways they are useful models for testing the effects of potential anti-cancer agents on these pathways. PC-3 cells are a model of aggressive androgen-unresponsive PCa^5^, while MDA-MB-231 cells lacking expression of receptors for estrogen (ER) and progesterone (PgR) and lacking amplification of human epidermal growth factor 2 (HER-2) have been used as a model of triple-negative breast cancer (TNBC)^6^.

The increased understanding of cellular signaling pathways known to be deregulated in cancer has resulted in considerable progress in generation of targeted therapies. However, despite this progress, in many cases the progression of cancer continues to a fatal stage, and the need to develop additional means to control development and progression of cancer remains critical. An equally important consideration has been to devise strategies for achieving chemoprevention of various cancers and to understand the mechanism of action of various agents currently proposed as chemopreventive agents. Since medicinal plants have been used for centuries to treat a variety of diseases and have been the initial source of many medicines in modern use, there has been intense interest in finding compounds that interfere with cell signaling pathways in order to develop improved and effective disease treatments. Considerable effort has also centered around identifying agents present in diets or other plants that could serve as chemopreventive or chemotherapeutic agents for various type of diseases including cancer with the advantage that they may have minimal or no unwanted toxicity (see, e.g.,^7^,^8^,^9^,^10^,^11^). Along these lines, one of the first studies demonstrated the activity of polyphenolic compound EGCG (epigallocatechin-3-gallate) present in green tea extract to influence the growth of various cancers as well as its chemopreventive properties^11^. Over time, several similar dietary derived plant-based agents (such as delphinidin, lupeol, fisetin, and pomegranate) have been examined with promising results indicating their usefulness as chemopreventive and/or therapeutic agents by themselves alone or in combination with other drugs (see e.g.^10^,^12^,^13^,^14^). It is noteworthy that many of the dietary and plant agents studied thus far exert their effect on a large number of cellular pathways^11^,^15^. Establishing the molecular mechanisms of action of phytochemicals can provide the basis of their utility as nontoxic and effective chemopreventive and/or therapeutic agents for cancer^16^.

*Acacia hydaspica* R. Parker belongs to the family *Leguminosae* and the species is commonly found in Iran, India and Pakistan where it has generally been used as fodder, fuel and wood^17^. The bark and seeds are the source of tannins. However, species of the *Leguminosae* family, particularly the genus *Acacia,* possess various bioactive compounds which may be useful as phyto-pharmaceuticals. Bioactive compounds such as (+)-catechin, (−)-epicatechin, (−)-epicatechin-3-*O*-gallate, epigallocatechin-3-*O*-gallate, quercetin, and (+)-cyanidanol have been isolated from different *Acacia* species^15^. Compounds identified from *Acacia* species are known to modulate various signaling pathways in breast and prostate cancer^18^,^19^. However, these studies have generally utilized the total extracts from various catechin sources reflecting effects of combined activities of several compounds rather than of a single compound. In the present work, we have examined the effects of some of the individual, purified bioactive compounds isolated from *Acacia hydaspica* R. Parker. For this, we subjected *A. hydaspica* to bioassay-guided fractionation which led us to the identification of 7-*O*-galloylcatechin (GC), catechin (C), methyl gallate (MG) and catechin-3-*O*-gallate (CG) as cytotoxic agents. The purified compounds (GC, C, CG and MG) isolated from *A. hydaspica* were investigated for their effects on certain cell signaling pathways and cell survival in PC-3 and MDA-MB-231 cell lines as models of prostate and breast cancer. The results provide, for the first time, evidence that *A. hydaspica* compounds (AHCs) could potentially serve as chemopreventive/therapeutic agents against cancer.

## Results

### Identification of isolated compounds

**Figure 1:**
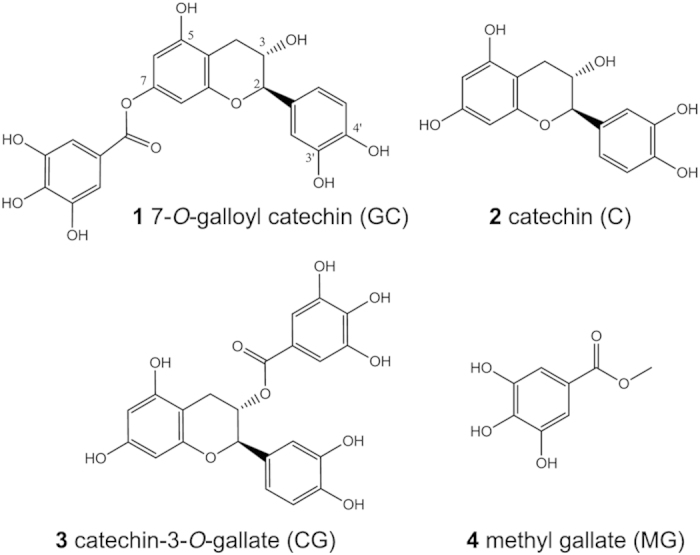
Structures of isolated compounds from *A. hydaspica* R. Parker. Chemical structures of the various compounds purified from *A. hydaspica* are shown.

The aerial parts (bark, twigs, and leaves) of *A. hydaspica* were collected and subjected to extensive extraction and fractionation as described in the Materials and Methods section. Diagrammatic illustration of the process is shown in Supplementary Fig. 1. Structures of purified compounds were elucidated by 1D and 2D NMR and mass spectrometric analysis (ESI and APCI). The compounds isolated from *A. hydaspica* were identified by comparison of the physical data with those reported previously. The data indicated purification of the polyphenol methyl gallate (MG)^20^ and three flavan-3-ols (+) 7-*O*-galloyl catechin (GC)^21^, (+) catechin (C)^22^,^23^, and (+) catechin-3-*O*-gallate (CG)^24^; chemical structures of the isolated compounds are shown in Fig. 1.

### Effects of AHC compounds on cell viability

**Figure 2:**
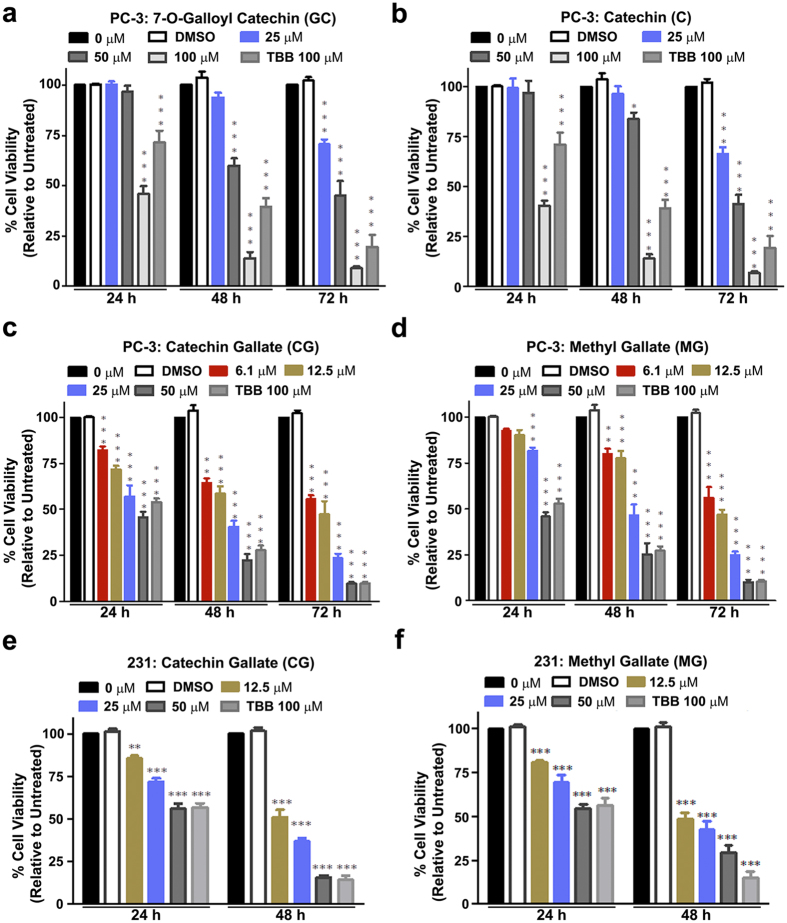
Cell viability in cells treated with AHC compounds. (**a–d**) Prostate cancer PC-3 cells were treated with varying concentrations of (**a**) 7-*O*-galloyl catechin, (**b**) catechin, (**c**) catechin gallate, or (**d**) methyl gallate for 24, 48 and 72 h as indicated. TBB was included as a positive control and DMSO was included as a negative control at a concentration equivalent to 100 μM AHC compound. Cell viability was determined relative to the untreated (0 μM) cells. (**e**,**f**) Breast cancer MDA-MB-231 cells were treated with varying concentrations of (**e**) catechin gallate, or (**f**) methyl gallate for 24 or 48 h, as indicated. All other conditions are as describe for (**a**–**d**). Data are presented as mean ± SEM (n = 3). Data analyzed by two-way ANOVA with Bonferroni post-test. The asterisks ^*^, ^**^, ^***^indicate significant difference at *p *<* 0.05, p* < *0.001* and *p* < 0.0001, respectively, from the untreated control cells.

To investigate the effects of AHCs on cancer cell viability, PC-3 (an androgen-independent prostate cancer cell line) and MDA-MB-231 (a triple negative breast cancer cell line) were chosen as two representative cell lines. PC-3 and MDA-MB-231 cells were grown in 96-well plates and treated with AHCs at concentrations varying from 0 μM to 100 μM. To assess the effects of these compounds, we employed the Cell Titer 96® Aqueous One assay. The assay involves conversion of a MTS tetrazolium compound to a colored formazan product whose absorbance is directly proportional to the number of metabolically active cells and thus indirectly measures cell growth. As shown in Fig. 2, GC, C, CG and MG retarded PC-3 cell growth in a dose- and time-dependent manner as compared with untreated or DMSO treated control groups. The relative viability after 24 h of treatment with 100 μM doses of GC and C was 45.9 ± 3.7 and 40.5 ± 2.5%, respectively. The viability of cells decreased further after 48 h and 72 h of treatment. Similarly, the percentages of viable cells after 24 h treatment with 50 μM CG and MG were 45.5 ± 3.1% and 45.8 ± 2.3%, respectively, and viability continued to decrease through 72 h of treatment (Fig. 2). The well-known CK2 inhibitor 4,5,6,7-tetrabromobenzotriazole (TBB) was used as a positive control; however, it reduced viability in PC-3 cells to a lesser extent than the AHCs at similar concentrations.

The cell viability studies demonstrated that CG and MG were the most potent anti-proliferative compounds in MDA-MB-231 cells, producing both dose- and time-dependent effects (Fig. 2). Cell viability with 50 μM CG was 56.0 ± 3.0% and 15.5 ± 1.2%, following 24 h and 48 h of treatment, respectively. The percent survival after 24 h and 48 h treatment of MDA-MB-231 cells with 50 μM MG was 54.6 ± 2.8% and 29.5 ± 4.0%, respectively. Lower doses (12.5 and 25 μM) also showed some cytotoxicity at 48 h of treatment. The effects of 100 μM TBB on cell viability were similar to those observed with 50 μM CG and MG. GC and C had relatively little effect on these cells (data not shown).

### Detection of cell death by morphological analysis

In order to determine the role of apoptosis in cell growth inhibition by AHCs, morphological changes in PC-3 cells and MDA-MB-231 cells were examined by phase contrast microscopy (Supplementary Fig. 2). PC-3 cells were treated with 100 μM of GC and C, and 50 μM of CG and MG; MDA-MB-231 cells were treated with 50 μM of CG and MG. Phase contrast microscopy showed that treatment with AHCs for 24 and 48 h in both cell lines resulted in low cell confluence and membrane blebbing, indicative of apoptosis. Moreover, floating cells indicated that AHC treatment resulted in reduced adherence. Untreated or DMSO-treated (at volume equivalent to that for 100 μM AHC) cells were attached to the culture plates with greater than 90% confluence under the same conditions as those for AHC treated cells (Supplementary Fig. 2).

**Figure 3:**
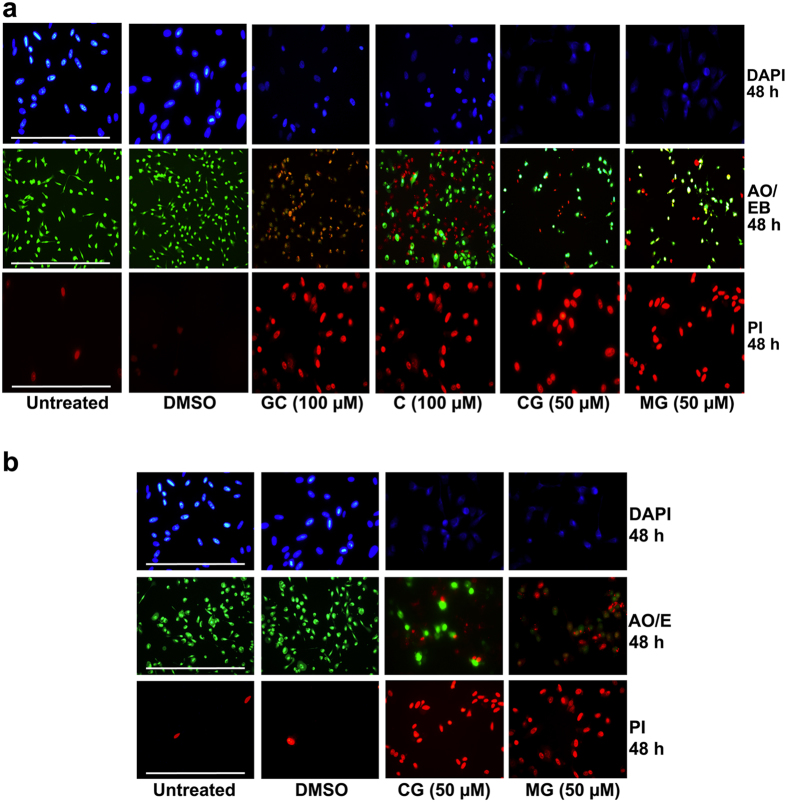
Detection of apoptosis by fluoresence microscopy in cells after treatment with AHC compounds. (**a**) PC-3 cells were treated with GC, CG or MG for 48 h, as indicated below the panels. Cells were stained with DAPI, acridine orange and ethidium bromide (AO/EB), or propidium iodide (PI) as indicated on the right side of the panels. (**b**) MDA-MB-231 cells were treated with CG or MG for 48 h as indicated below the panels. Cells were stained with DAPI, acridine orange and ethidium bromide (AO/EB), or propidium iodide (PI) as indicated on the right of the panels. Images of cells were captured at 200-fold magnification. Scale bar is 100 μm.

Fluorescence microscopy analysis of DAPI stained cells was undertaken to study nuclear alterations and apoptotic body formation, both of which are also features of apoptosis^25^. Cells treated with AHCs exhibited apoptotic morphology in both types of cancer cells under investigation. Morphological changes characterized by cytoplasmic and nuclear shrinkage, chromatin condensation and apoptotic body formation were observed in response to treatment of both PC-3 (Fig. 3a) and MDA-MB-231 (Fig. 3b) cells with AHCs. In the untreated or DMSO treated PC-3 and MDA-MB-231 cells, the stained nuclei were rounded and homogeneously stained with DAPI, whereas treated cancer cells from both cell lines showed an altered nuclear DNA staining pattern with condensed chromatin and apoptotic bodies that are hallmarks of early and late apoptosis.

The results of acridine orange (AO) and ethidium bromide (EB) staining of cells treated with AHCs are shown in Fig. 3. AO is a cell permeable fluorescent dye and stains nuclear DNA in both live and dead cells, whereas EB is a fluorescent dye that only stains nuclear DNA in cells that have lost their membrane integrity^26^. We observed that after AO/EB staining viable cells were uniformly stained green, early apoptotic cells were stained greenish yellow or displayed green yellow fragments, late apoptotic cells were stained orange or displayed orange fragments, and necrotic cells showed orange to red fluorescing nuclei with no indication of chromatin fragmentation. In PC-3 cells, treatment with GC (100 μM) showed late apoptotic cells that were densely stained as orange. Treatment with C (100 μM) showed live cells with initial phases of apoptotic nuclei appearing as “viable apoptotic” (VA) as indicated by green chromatin which was highly condensed or fragmented with some cells undergoing cell death. Dead cells also showed apoptotic nuclei as chromatin appeared to be fragmented indicating cells to be “nonviable apoptotic” (NVA). CG and MG were more effective in inducing cell death as compared with GC and C in PC 3 cells, and the mode of cell death was apoptosis indicated by highly fragmented and condensed chromatin which appeared bright orange (Fig. 3a). Similar results were obtained for MDA-MB-231 cells treated with various compounds (Fig. 3b).

Propidium iodide (PI) is a red fluorescent dye that is impermeable to the cell membrane of viable cells, and is used to stain the DNA of both necrotic and apoptotic cells. After 48 h of AHC treatment, untreated or DMSO treated cells showed no significant change in their viability as indicated by the very low number of cells staining with PI. In contrast, all tested compounds induced significant cell death as indicated by PI staining in PC-3 and MDA-MB-231 cells (Fig. 3).

### AHCs induce loss of clonal survival

**Figure 4:**
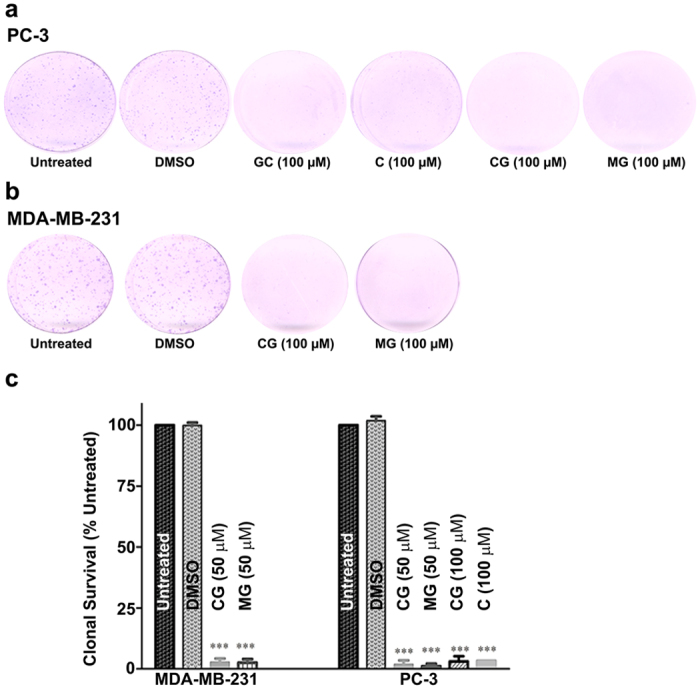
Clonogenic survival assays following treatment with AHC compounds. (**a**,**b**) PC-3 (**a**) or MDA-MB-231 (**b**) cells were treated with AHC compounds or equal dilution DMSO (as indicated below the plate images). After 48 h, the compounds were removed and cells seeded at a density of 1000 cells for PC-3 and 500 cells for MDA-MB-231 on 35 mm plates. After 7 days of growth, the cells were stained with crystal violet and the stained plates scanned. Representative plates are shown. (**c**) Crystal violet stained colonies were quantified as described in the “Materials and methods” section. The percent inhibition of PC-3 and MDA-MB-231 colony formation relative to untreated control is graphed. Data are presented as mean ± SEM of at least 3 independent experiments. Data analyzed by one-way ANOVA with Bonferroni post-test. Asterisks ^***^indicate significant difference at *p* < 0.0001 relative to untreated control.

In order to assess the survival and proliferative capacity of the PC-3 and MDA-MB-231 cells following treatment with AHCs, clonal survival assays were carried out. In these assays, the AHCs were added to the cells and then removed after a 48 h period of treatment. The cells were counted and re-plated in complete media as described under Materials and Methods. After 7 days of growth, the cells were stained with crystal violet and colonies of 50 or more cells were counted. Representative stained colony plates are shown in Fig. 4; also depicted in Fig. 4 is the plot showing quantitation of the survival data for PC-3 and MDA-MB-231 cells. As shown, MDA-MB-231 and PC-3 cell colony numbers were significantly reduced (p < 0.001) compared to untreated or DMSO treated control groups. In PC-3 cells, cell shrinkage and a shift from stellate to a rounded appearance was more obvious as compared to MDA-MB-231 cells. We also observed small scattered cells unable to colonize and reduced colony size in both cancer cell types.

### Signaling pathways and survival proteins modulated by AHC treatment

**Figure 5:**
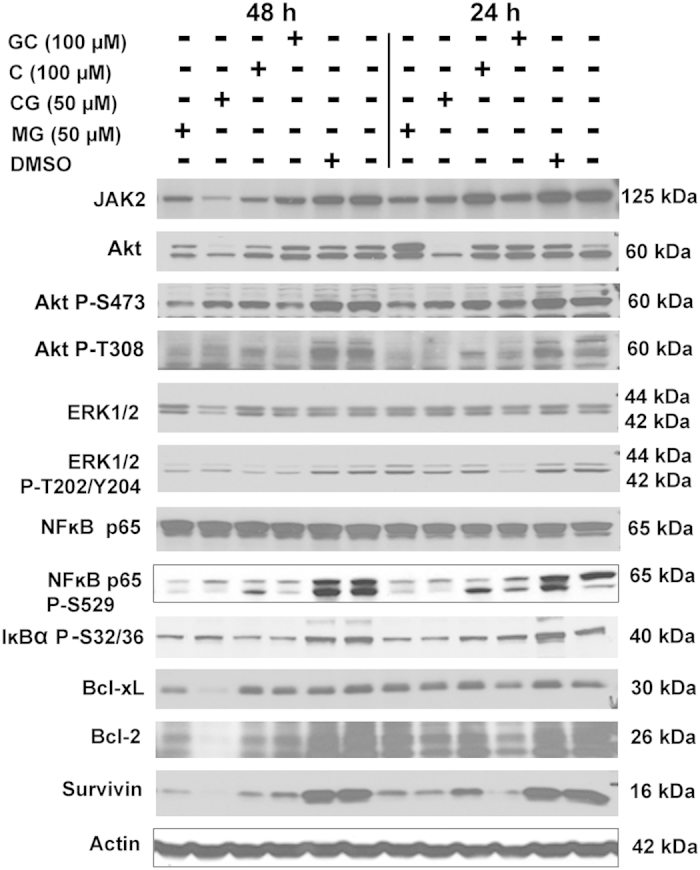
Western blot analyses following treatment of PC-3 cells with AHC compounds. Western blot analysis of cellular lysates prepared from PC-3 cells treated with 7-*O*-galloyl catechin (GC), catechin (C), catechin gallate (CG), methyl gallate (MG), or equal dilution of DMSO was performed. Treatments are indicated above the blots. The protein detected is indicated to the left of each blot, and the size of protein detected is indicated to the right of each blot. The quantitation of the data is shown in Supplementary Table 2. Cropped blots are shown. Full sized blots are included in Supplementary Fig. 3. All gels and blots were run under the same experimental conditions as described in Methods.

**Figure 6:**
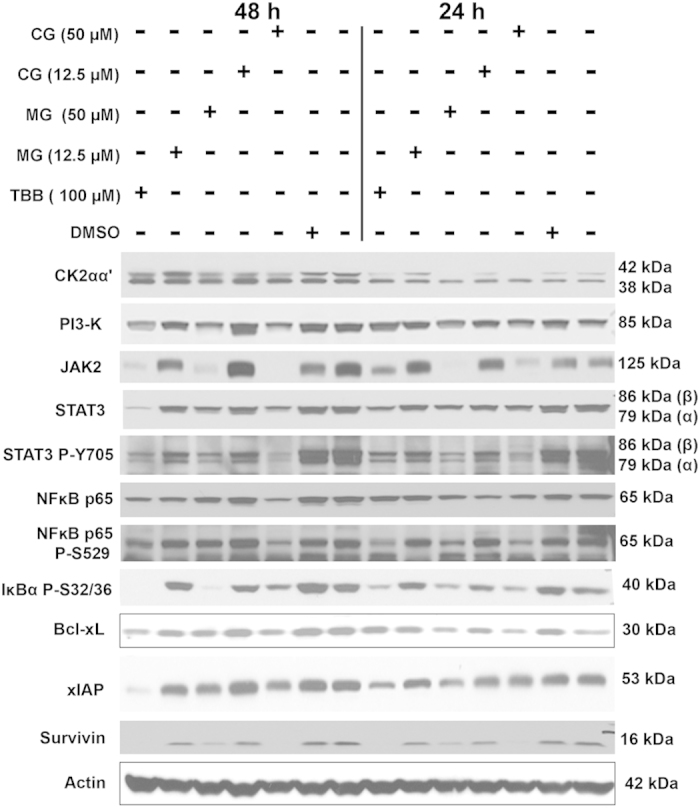
Western blot analyses following treatment of MDA-MB-231 cells with AHC compounds. Western blot analysis of cellular lysates prepared from MDA-MB-231 cells treated with 2 concentrations each of catechin gallate (CG) and methyl gallate (MG), or dilution of DMSO representing the highest concentration was performed. Treatments are indicated above the blots. The protein detected is indicated to the left of each blot, and the size of protein detected is indicated to the right of each blot. The quantitation of the data is shown in Supplementary Table 3. Cropped blots are shown. Full sized blots are included in Supplementary Fig. 4. All gels and blots were run under the same experimental conditions as described in Methods.

Many of the chemopreventive agents are known to exert moderate inhibitory effects on diverse survival signaling pathways (e.g.,^9^). Thus, we decided to examine the effects of AHCs on certain survival signaling pathways in prostate and breast cancer cells. For these studies, we performed western blot analyses of lysates from PC-3 cells treated with 100 μM GC and C, and 50 μM CG and MG, and from MDA-MB-231 cells treated with two doses of compounds CG and MG (12.5 μM and 50 μM) for 24 or 48 h. For both cell lines, the signals analyzed were CK2α, CK2α′, PI3K, JAK2, Akt, Akt P-Ser473, STAT3, STAT3 P-Tyr705, ERK 1/2, ERK 1/2 P-Thr202/P-Tyr204, NFκB p65, NFκB p65-P-Ser529, IκBα P-Ser32/36, Bcl-2, Bcl-xL, xIAP, and survivin. The data shown in Fig. 5 (for PC-3 cells) and Fig. 6 (for MDA-MB-231 cells) are only for the signals that demonstrated changes in response to treatment with various agents (signals that were unchanged are not included); however, the quantitation of all signals (responsive as well as unresponsive) at 24 h and 48 h of treatment is shown in Supplementary Tables 2 and 3.

Polyphenolic compounds such as EGCG and resveratrol are known to exert an inhibitory effect on protein kinase CK2 in prostate cancer cells^27^. Thus, we examined the effects of these AHCs on CK2 and certain other signals in both prostate and breast cancer cells. Treatment of PC-3 cells with AHCs did not alter the expression levels of CK2α or α′ (Supplementary Table 2). Further, addition of AHCs to kinase activity assays did not lead to a dose dependent effect using purified CK2 (data not shown). However, in breast cancer cells, there was a dose-dependent effect of CG and MG on CK2α level but not of CK2α′ (Supplementary Table 3 and Fig. 6). PI3K signals in PC-3 cells were also not affected by various AHCs, but CG and MG exerted a dose-dependent effect in breast cancer cells (Supplementary Table 3). JAK2 was significantly inhibited by the AHCs tested in PC-3 cells and both CG and MG were potent inhibitors at 50 μM concentration in MDA-MB-231 cells. Akt was not inhibited by GC and C but showed significant reduction in the presence of CG and MG in prostate cancer cells, and significant reductions were also noted in the Akt-P-Ser473 and P-Thr308 signals (Supplementary Table 2). However, these signals did not show any change in response to CG and MG in breast cancer cells (Supplementary Table 3). STAT3 and STAT3-P-Tyr705 were unresponsive to the AHCs tested in PC-3 cells (Supplementary Table 2), whereas these signals showed significant reductions in MDA-MB-231 cells in response to the AHCs tested (Supplementary Table 3). CG at 50 μM had a moderate effect on ERK 1/2 but the corresponding ERK 1/2-P-Thr202/P-Tyr204 demonstrated a dramatic decrease in response to all the AHCs tested in PC-3 cells (Supplementary Table 2). On the other hand, the breast cancer cells did not demonstrate any significant effect on these two signals in response to CG and MG (Supplementary Table 3). NFκB p65 was unaffected by the various AHCs in PC-3 but there was a significant reduction in the p65-P-Ser529 signal; the IκBα P-Ser32/36 signal was markedly reduced in the PC-3 cells in response to the AHCs and was analogous to the reduction of p65 phosphorylation-specific signal (Supplementary Table 2). In the MDA-MB-231 cells, reduction of p65-P-Ser529 was concordant with that of the p65 in the presence of 50 μM CG or 50 μM MG (Supplementary Table 3). The effect on Bcl-2 in PC-3 cells was apparent in the presence of CG and MG (both at 50 μM concentrations) but not GC and C (at 100 μM) (Supplementary Table 2). Bcl-xL signal was reduced and demonstrated varying levels of response to different AHCs in both the prostate and breast cancer cells. The AHCs did not produce a significant change in level of xIAP in PC-3 or MDA-MB-231 cells, as compared to the change induced by TBB treatment. Finally, all the AHCs tested produced a significant loss of survivin signal in both the PC-3 and MDA-MB-231 cells (Supplementary Tables 2 and 3). These results suggested that AHCs result in varied effects on diverse survival signals in the prostate and breast cancer cells dependent on the type and dose of AHC.

## Discussion

Several studies have indicated a reduced risk of developing prostate and breast cancer in humans consuming diets rich in fruits and vegetables; the principal plant derived agents thought to provide protection against cancer are flavonoids and dietary fiber^7^,^28^,^29^. Flavonoids have been demonstrated to exert their effects on a variety of biological functions including inhibiting the cell cycle, reducing oxidative stress, augmenting the efficacy of detoxification enzymes, inducing apoptosis, and stimulating the immune system. These inherent properties of flavonoids categorize them as a class of beneficial compounds possessing health-promoting and disease-preventing effects, including potential efficacy in cancer prevention^30^,^31^. In the present study, we have attempted to study the mechanism of action of the flavanols isolated from *A. hydaspica* on human androgen-independent prostate cancer (PC-3) and triple negative breast cancer (MDA-MB-231) cell lines as models.

**Figure 7 f7:**
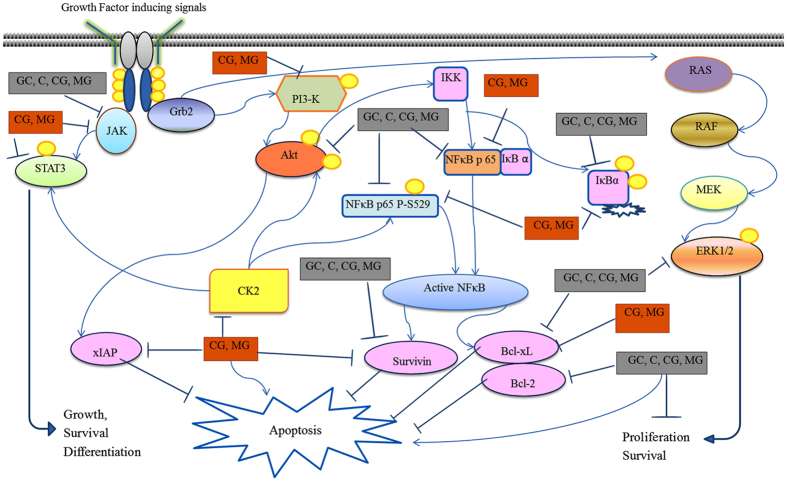
Proposed signaling pathways and anti-apoptotic proteins expression affected by compounds isolated from *A. hydaspica.* A diagram of the effects of AHC compounds, in PC-3 and MDA-MB-231 cells as detected by western blot analyses is depicted. Effective compounds against PC-3 cells are depicted in grey boxes. Effective compounds against MDA-MB-231 cells are depicted in red boxes. Arrows indicate activation or stimulation. Blocked lines indicate inhibition. Yellow circles indicate phosphorylation.

Our results based on a viability assay indicated that GC, C, CG and MG induced growth arrest in PC-3 cells, however MDA-MB-231 cells were responsive to only CG and MG. Similarly, GC, C, CG and MG blocked cell proliferation and clonogenic survival in prostate cancer cells, whereas CG and MG inhibited breast cancer cell growth. The data also suggested that various AHCs induced apoptosis in both PC-3 and MDA-MB-231 cells. Although inhibition of cell growth and induction of apoptosis were sometimes associated with modulation of different signaling pathways in breast and prostate cancer cells, our data, in general, suggest that AHCs exerted influence on various signaling factors such as PI3-K, JAK2, Akt-1, STAT3, MAPK (ERK1/2, pERK1/2), NFκB, Bcl-xL and survivin (summarized in Fig. 7).

We observed direct inhibition of CK2α protein expression in MDA-MB-231 cells by 50 μM doses of CG and MG. Interestingly, although we observed no effect on CK2 protein expression in PC-3 cells following AHC treatment, the CK2 specific phosphorylation site NFκB p65 P-Ser529 was significantly inhibited by AHC treatment in both cell lines. The *A. hydaspica* AHCs further acted to decrease activation of NFκB by inhibiting phosphorylation and degradation of IκBα P-Ser32/36 in PC-3 and MDA-MB-231 cells. These findings suggest that inhibition of NFκB pathway activation may be one of the mechanisms by which AHCs induced growth arrest in MDA-MB-231 and PC-3 cells. Aberrant activation of NFκB is associated with initiation or acceleration of tumorigenesis and enhances resistance to chemotherapy in cancer cells^32^. These observations hint that treatment with AHCs could potentially exert a significant chemopreventive activity.

CK2 is also known to regulate Akt-1 and phosphatidylinositol 3 kinase (PI3-K)^33^,^34^. Our results indicated that in PC-3 cells GC, C, CG and MG treatment decreased the level of phosphorylated forms of Akt-1 (Ser473 and Thr308); CG and MG also significantly decreased Akt total protein expression in PC-3 cells. Akt protein phosphorylation at Ser473 and Thr308 sites is necessary for Akt-mediated transcription. In MDA-MB-231 cells, the expression of phosphorylated and total Akt protein remained unchanged after treatment; this might be due to the presence of active PTEN, as PTEN protein expression was unchanged upon treatment with CG and MG in the MDA-MB-231 cells (data not shown). Other pro-survival pathways known to be active during progression of cancer include MAPK (ERK, JNK, p38) and STAT3. These pathways are candidates for therapeutic targeting^35^. Interestingly, CK2 is also involved in activation of the JAK-STAT signaling pathway^36^,^37^. We observed that in PC-3 cells JAK2 protein expression was significantly inhibited with little or no change in STAT3 phosphorylation. In these cells there was also a marked inhibition of MAPK, i.e., ERK 1/2 phosphorylation, which could be a possible mechanism for contributing to induction of cell death in PC-3 cells.

Evaluation of the mechanism of cell death induced in both prostate cancer (PC-3) and a breast cancer cell line (MDA-MB-231) by various active AHCs indicated that the primary mode of cell death was via apoptosis. NFκB activation pathway is known to regulate various signals in the apoptotic machinery^38^. We have demonstrated that AHCs significantly suppress cell proliferation and sensitize cells to apoptosis induction which in PC-3 cells is associated with decreased expression of Bcl-2 and Bcl-xL, whereas in MDA-MB-231 cells Bcl-xL expression was reduced significantly (p < 0.001) without a significant change in Bcl-2 expression. This latter result might be due to the highly significant decrease in the phosphorylation NFκB p65 in PC-3 cells since the level Bcl-2 is regulated by NFκB in prostate cancer cells^39^. Our results also demonstrated a significant inhibition of survivin expression in both PC-3 and MDA-MB-231 cells treated with AHCs. Survivin, an inhibitor of apoptosis, is highly expressed in most cancers and closely related to multiple-drug resistance, increased tumor recurrence, and reduced survival of patients^40^,^41^,^42^. Thus, the current findings demonstrating significant inhibition of survivin expression by AHCs provides further support for their potential role in chemoprevention or therapeutic activity.

Taken together, our results strongly suggest that CG and MG have effects on a variety of cell signaling pathways and may be effective as chemo-preventive agents against triple negative breast cancer. Likewise, treatment of human prostate cancer cells with GC, C, CG and MG induced apoptosis and resulted in effects on a variety of survival signals suggesting the potential of these compounds as chemopreventive agents for prostate cancer. In summary, our results suggest that compounds from *A. hydaspica* might be promising candidates for molecular target-based cancer prevention and adjuvant therapy. The novel information generated in the present work on the compounds derived from *A. hydaspica* provides important preliminary information that may be useful in designing further studies to ascertain the chemo-preventive and chemotherapeutic efficacy of these compounds.

## Materials and Methods

### Plant collection, extraction and isolation of compounds

The aerial parts (bark, twigs, and leaves) of *A. hydaspica* were collected from the Kirpa area of Islamabad, Pakistan, and after identification a voucher specimen (0642531) was assigned prior to submission to the Herbarium of Pakistan, Museum of Natural History, Islamabad.

Shade dried aerial parts (bark, twigs, and leaves) of *A. hydaspica* were ground in a Wiley mill of 60 mesh size sieve to a fine powder. Fractionation of compounds from this material was carried out as shown in Supplementary Fig. 1. Plant powder (3 kg) was extracted thrice with 7 L of crude methanol at 25 °C for 72 h. The extract was filtered through Whatman No. 1 filter paper, concentrated under vacuum using a rotary evaporator (Buchi, R114, Switzerland) at 40 °C, and 472 g of *A. hydaspica* crude extract (AHM, 15.73%) was obtained. For initial fractionation of different plant constituents of varying polarities, the crude methanol extract (12 g) was suspended in distilled water and partitioned consecutively with *n-*hexane (3 × 250 ml), chloroform (3 × 250 ml), ethyl acetate (3 × 250 ml), and *n-*butanol (3 × 250 ml). The resultant fractions were dried using a rotary evaporator. The following yields were obtained: *n*-hexane (AHH, 5%), ethyl acetate (AHE, 28%), chloroform (AHC, 2%), *n*-butanol (AHB, 42%). The crude methanol extract (AHM) and aqueous extract (AHA, 8% yield) were also obtained. The structures of various compounds isolated by this procedure are shown in Fig. 1.

### Bioassay Guided Isolation of active fractions and compounds

The hexane and butanol soluble fractions of *A. hydaspica* that showed the most potent effects on cell viability using the Cell Titer 96 Aqueous One cell proliferation assay (Promega Corp., Madison, WI) were chosen for this study (Supplementary Table 1). The fractions (AHE and AHB) were subjected to vacuum liquid chromatography (VLC) using silica gel and reverse phase C18 systems, respectively. Briefly 10 g of AHE was dissolved in dichloromethane (DCM), mixed with Celite (Diatomaceous Earth) and dried with a rotary evaporator. The dried extract sample was loaded onto silica gel using a glass column packed with silica (200–400 mesh) and attached to a vacuum line source. The column was eluted with DCM followed by a DCM-methanol mixture with increasing polarity gradient. Fractions were dried using a rotary evaporator and similar fractions were pooled according to their TLC patterns (Silica gel 60 F_254_ plates, MERCK) into 12 fractions. Three major phenolic fractions (EF4-EF6) were selected according to their ^1^HNMR spectra similarity and combined and subjected to flash liquid chromatography using a Combi-flash Teledyne ISCO system with normal phase silica. The sample was purified using a 40 g Redisep column with a gradient of DCM to methanol, with a flow rate of 15 ml/min. The fractions were pooled into 10 samples according to their TLC and ISCO chromatogram peaks. Each fraction was tested for cytotoxic activity against PC-3 and MDA-MB-231 (Supplementary Table 1). ^1^HNMR of active fractions indicated the presence of methyl gallate (MG, 100mg, white powder) and catechin (C, 100 mg, light yellow powder). Active fraction IF 9 was further purified using Sephadex LH-20 (Pharmacia Co., Ltd) with methanol as an eluent, and pure 7-O-galloyl catechin (GC, 500 mg, light green powder) was obtained.

The butanol soluble fraction (AHB) was fractionated by VLC using C18 and eluted with a mixture of H_2_O:MeOH as described above. Fractions were dried using rotary evaporator and similar fractions were pooled according to their TLC Rf values (Silica gel 60 F_254_ plates, MERCK) into 10 fractions. These fractions were tested for cytotoxicity, and the active fraction BF1 was further purified on Sephadex LH 20 to yield 8 pooled fractions (BSF1-BSF8). Active cytotoxic fraction BSF-4 was further purified using semi-prep HPLC (Agilent 1260 series) with a Grace Vision HT C18 column (10 μm; 10 × 250 mm, USA). The fractions were purified using a gradient of A:water and B:acetonitrile over 40 minutes at 3ml/min (0–5 min with 85% A, 5–25 min for 15 to 100% B, and isocratic at 100% B through 40 min) and yielded catechin-3-*O*-gallate (CG, 150 mg, light yellow amorphous powder).

### Identification of compounds

The ^1^H- and ^13^C-NMR spectra for all compounds were obtained on a Varian 600 MHz NMR at 25 °C. Spectra of all compounds were obtained in methanol-d4 and DMSO-d6. Detailed analysis of resolution-enhanced spectra (peak picking, integration, multiplet analysis) was performed using Varian NMR and ACD/NMR processor Academic Edition software. The NMR spectra and chemical shifts of isolated compounds were compared with published data.

### Cell culture

The human prostate cancer cell line PC-3 was purchased from American Type Culture Collection (CRL1435™, ATCC®, Manassas, VA), and the human breast cancer cell line MDA-MB-231 was obtained from David Potter (University of Minnesota). The MDA-MB-231 cell line was authenticated by STR profiling (IDEXX BioResearch). PC-3 cells were grown in RPMI 1640 (Invitrogen). MDA-MB-231 was grown in Dulbecco’s Modified Eagle’s Medium (DMEM)/F12 nutrient media (Hyclone, Thermo scientific). Media were supplemented with 1% penicillin/streptomycin, 10% fetal bovine serum, and 2 mM L-glutamine. Cells were cultured at 37 °C in a humidified atmosphere with 5% CO_2_.

### Cell viability assay

CellTiter 96^®^ Aqueous One assay was used to assess cell viability as previously described^43^. MDA-MB 231 and PC-3 cells were seeded into 96-well plates at 1.5 and 2.5 × 10^4^ cells/well, respectively. Treatments were performed in PC-3 cells using complete media and concentrations of 0 to 100 μM of GC, C, CG and MG for 24 h, 48 h, and 72 h. MDA-MB-231 cells were similarly treated with 0 to 50 μM of CG and MG for 24 h and 48 h. Controls included untreated and DMSO treated cells.

### Western blot analysis

SDS-page and western blot analyses were performed as in a previously described protocol with slight modifications^44^. Briefly, after 24 h and 48 h of treatments with AHCs at required doses, PC-3 and MDA-MB-231 cells were lysed in RIPA buffer supplemented with freshly added protease and phosphatase inhibitor cocktail 1:100 (Sigma) and protein concentration was determined by Bradford assay. Equal amounts of proteins were loaded using 4–12% Tris-glycine gels (NOVEX, Life Technologies, USA). Nitrocellulose membranes (Millipore) were blocked for 1 h with either 5% BSA (Sigma) or 5% nonfat dry milk (Bio-Rad, Cat.#170-6404) then incubated with primary antibody overnight at 4 °C. The following antibodies were used: CK2α (A300-197A) and CK2α′ (A300-199A) from Bethyl Laboratories; Stat 3 (#9139), phospho-Stat3 (Tyr 705, #9145), JAK2 (#3230), Akt (#2967), phospho-Akt (Ser473, #9271), phospho-Akt (Thr308, #9275), phospho-IκB Ser32/36 (#9246), Bcl-xL (#2762), ERK1/2 (#9102), phosphor-ERK1/2 (Thr202/Tyr204, #9101), XIAP (#2042), PI3K (#4292) from Cell Signaling Technology; Survivin (AF886) from R&D Systems; NFκB p65 (sc8008), phospho-NFκB p65 Ser529 (sc101751), and actin (sc1616) from Santa Cruz Biotechnology. Quantitation of protein bands was done by measuring band density from scanned films using Image J software. The density of the bands (normalized to actin) relative to that of the untreated control (designated as 1.00) were expressed as mean ± SEM of three independent experiments.

### Analysis of cellular morphological alterations

Analysis of cellular morphological alteration was performed at the end of each incubation period (24 h and 48 h) using phase contrast microscopy (Zeiss Axiovert).

### Analysis of apoptosis

Fluorescent staining was performed to study the effect of AHCs on cellular morphology. MDA-MB-231 cells were allowed to attach for 24 h to Poly-D-lysine coated cover slips, whereas PC-3 cells were seeded onto Matrigel coated cover slips. Cells at 70% confluence were treated as described below.

### Nuclear morphological analysis by DAPI

After treatment of both PC-3 cells and MDA-MB-231 cells with AHCs for 48 h, the monolayer of cells was washed with PBS and fixed with 4% paraformaldehyde (PF) for 10 min at room temp. Cells were washed with PBS and incubated for 10 min with Slow Fade Gold containing DAPI (Molecular Probes) protected from light. The cover slips were sealed onto slides with nail polish and stored at −20 °C until image collection by florescence microscopy.

### Acridine orange (AO)/ethidium bromide (EB) dual staining

PC-3 and MDA-MB-231 cells were treated with AHCs for 48 h. Cells were stained with AO/EB (4 μg/ml) for 5 min and images were immediately collected by fluorescence microscopy.

### Propidium iodide (PI) staining

Following treatment of PC-3 and MDA-MB-231 cells for 48 h, media was removed from the cells, PI staining solution was added (5 μg/ml), and cells were incubated at 37 °C for 15 min. Cells were washed with PBS and images were immediately collected by fluorescence microscopy.

### Clonal survival assay

PC-3 and MDA-MB-231 cells were collected after AHC treatments for 48 h, suspended in fresh medium, quantified, and 500 cells (MDA-MB-231) or 1000 cells (PC-3) were plated in triplicate into 35 mm cell culture dishes. After 7 days, colonies were stained with crystal violet as previously described^43^. Colonies were counted under dark field using a cubic colony counter (AO scientific). The number of cells in each colony was determined by phase contrast microscopy, and colony sizes were measured on images using Adobe Photoshop. Data are presented as mean colony number ± SEM relative to untreated controls (n = 3 independent experiments).

### Data analysis

Statistical analysis was carried out by using GraphPad Prism 5. Level of significance between different treatments groups relative to control was determined by one-way and two way analysis of variance (ANOVA) followed by Bonferroni test for between-group comparison. P < 0.05 was considered statistically significant. All data were presented as mean ± standard error of the mean (SEM) of three independent experiments.[Fig f7]

## Additional Information

**How to cite this article**: Afsar, T. *et al.* Growth inhibition and apoptosis in cancer cells induced by polyphenolic compounds of *Acacia hydaspica*: Involvement of multiple signal transduction pathways. *Sci. Rep.* **6**, 23077; doi: 10.1038/srep23077 (2016).

## Supplementary Material

Supplementary Information
