# Supplementary material for: Growth inhibition and apoptosis in cancer cells induced by polyphenolic compounds of Acacia hydaspica: Involvement of multiple signal transduction pathways
Source: Sci Rep. 2016 Mar 15;6:23077. doi: 10.1038/srep23077 (PMC4791679; doi:10.1038/srep23077)
Supplement: Supplementary Information [file srep23077-s1.pdf]

Supplementary Information

Growth inhibition and apoptosis in cancer cells induced by polyphenolic compounds of *Acacia hydaspica*: Involvement of multiple signal transduction pathways

Tayyaba Afsar[1], Janeen H.Trembley[2, 3], Christine E. Salomon[4], Suhail Razak[5] Muhammad Rashid Khan[1], Khalil Ahmed[2, 3*]

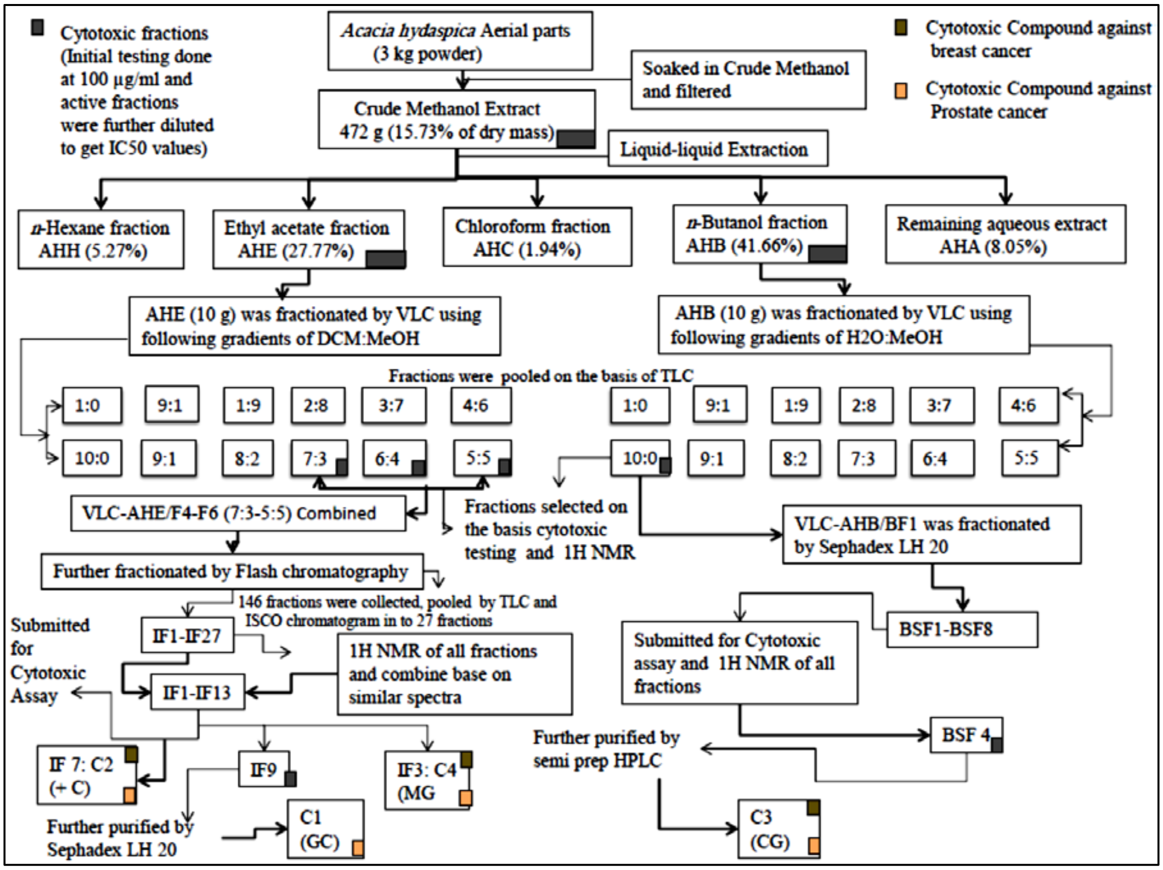

Supplementary Figure 1. **Schematic representation of extraction and isolation of compounds from *A. hydaspica.***

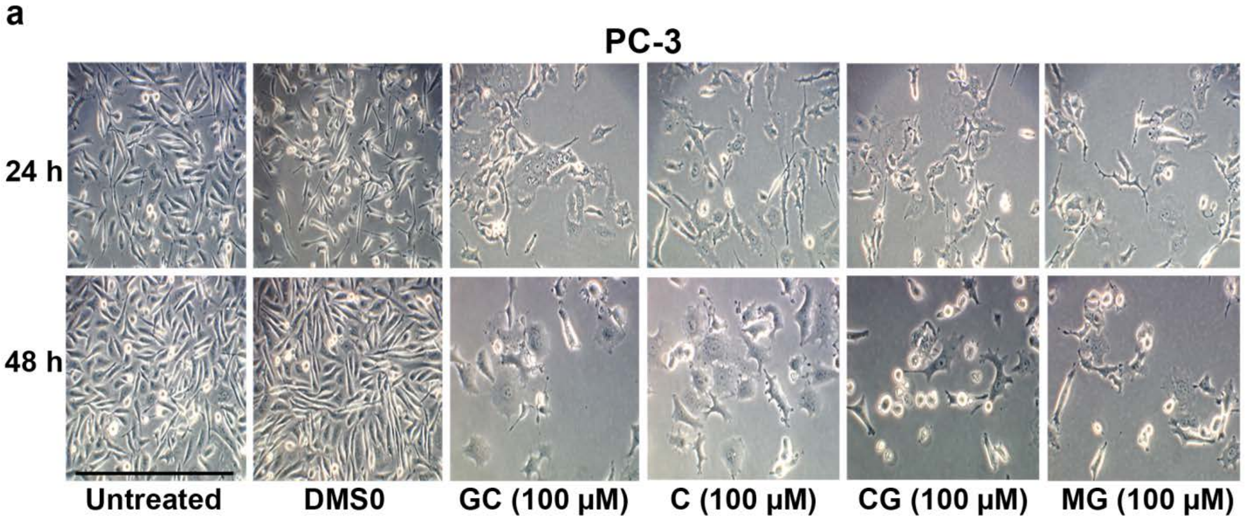

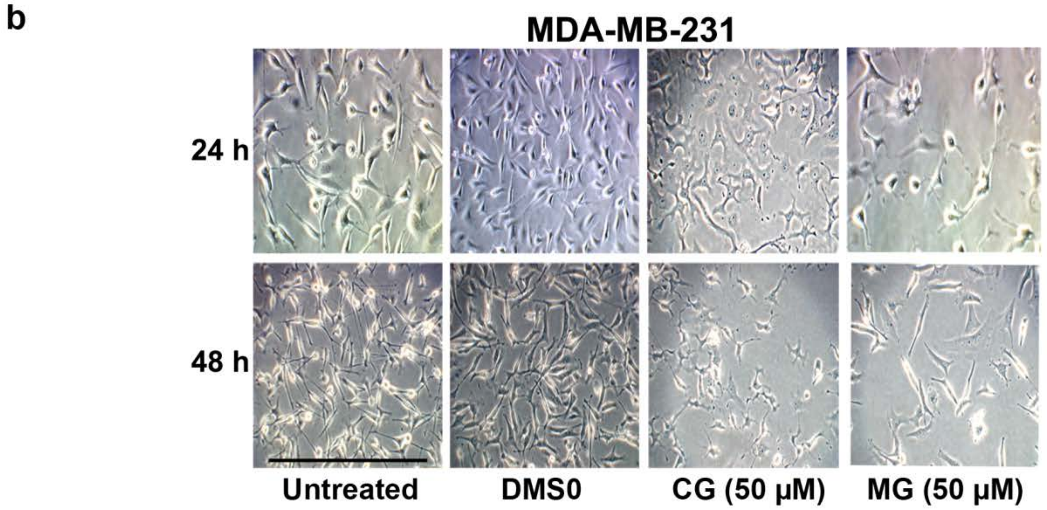

Supplementary Figure 2. Representative phase contrast images of AHC-treated PC-3 and MDA-MB-231 cells. (**a**); PC-3 cells were treated with GC (100 µM), C (100 µM), CG (50 µM), or MG (50µM). (**b**); MDA-MB-231 cells treated with 50µM dose of CG and MG. DMSO treatment was performed at a dilution equal to 100 µM for PC-3 and 50 µM for MDA-MB-231. Phase contrast images of cells were captured after 24 h and 48 h of AHC treatment at 200-fold magnification. Scale bar is 100 µm.

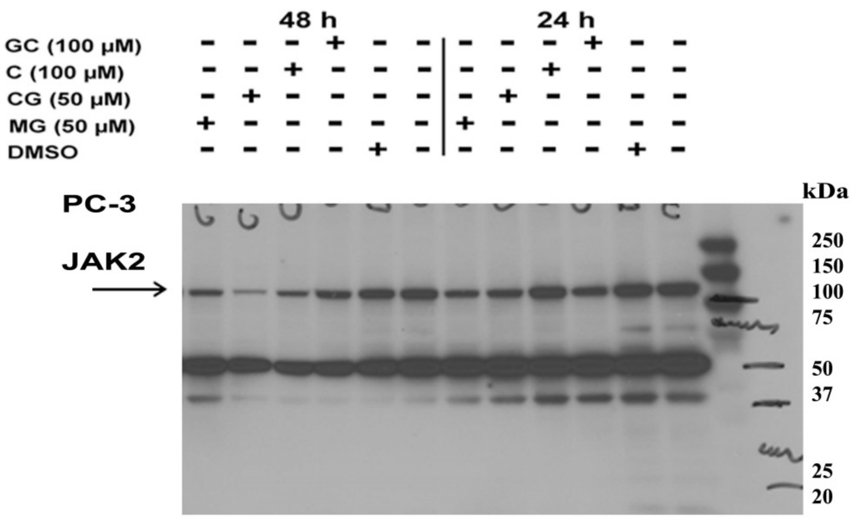
48 h
24 h
GC (100 µM)
C (100 µM)
CG (50 µM)
MG (50 µM)
DMSO
PC-3
JAK2
kDa
250
150
100
75
50
37
25
20

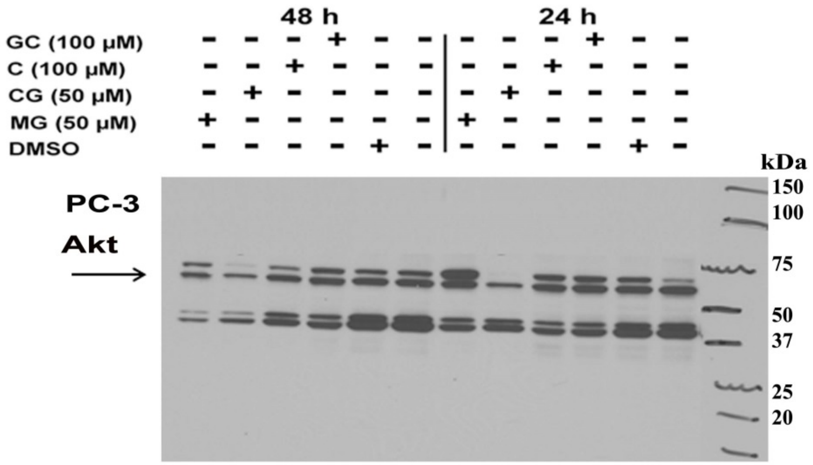
48 h
24 h
GC (100 µM)
C (100 µM)
CG (50 µM)
MG (50 µM)
DMSO
PC-3
Akt
kDa
150
100
75
50
37
25
20

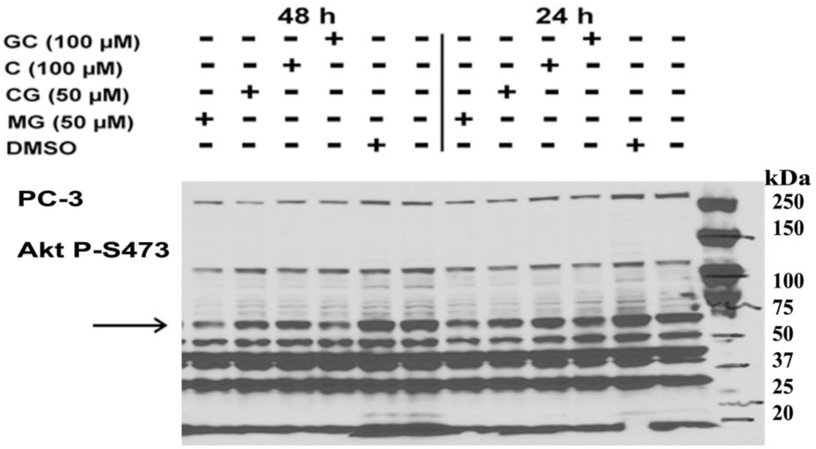
48 h
24 h
GC (100 µM)
C (100 µM)
CG (50 µM)
MG (50 µM)
DMSO
PC-3
Akt P-S473
kDa
250
150
100
75
50
37
25
20

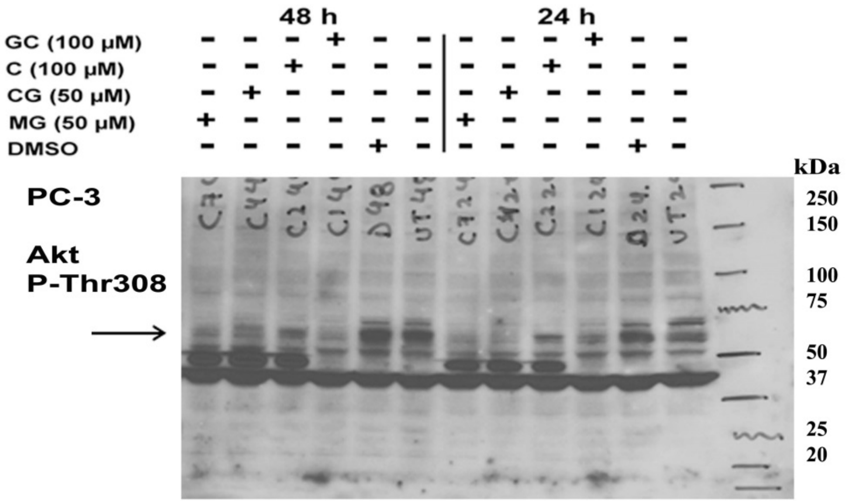
48 h
24 h
GC (100 µM)
C (100 µM)
CG (50 µM)
MG (50 µM)
DMSO
kDa
PC-3
Akt
P-Thr308
250
150
100
75
50
37
25
20

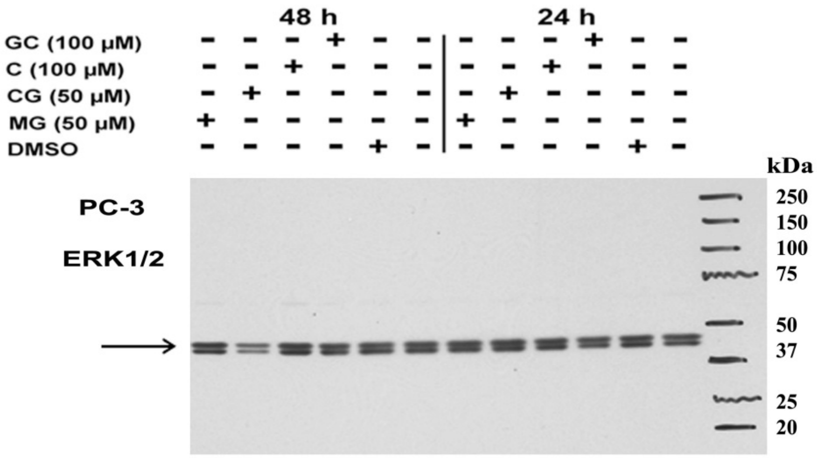
48 h
24 h
GC (100 µM)
C (100 µM)
CG (50 µM)
MG (50 µM)
DMSO
kDa
PC-3
ERK1/2
250
150
100
75
50
37
25
20

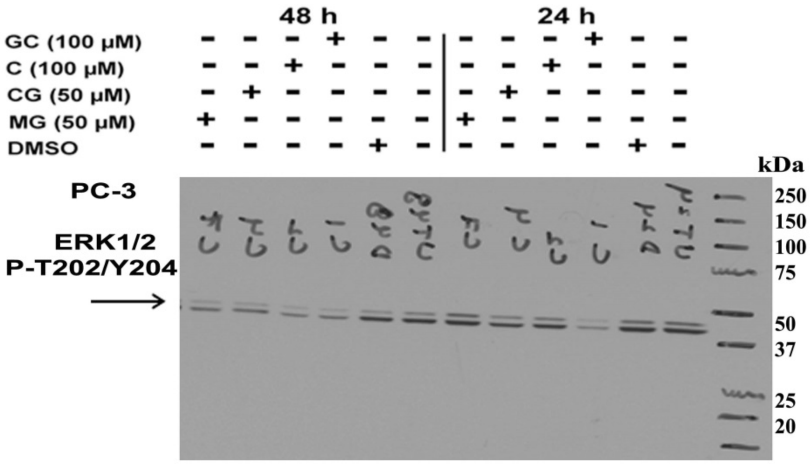
48 h
24 h
GC (100 µM)
C (100 µM)
CG (50 µM)
MG (50 µM)
DMSO
kDa
PC-3
ERK1/2
P-T202/Y204
250
150
100
75
50
37
25
20

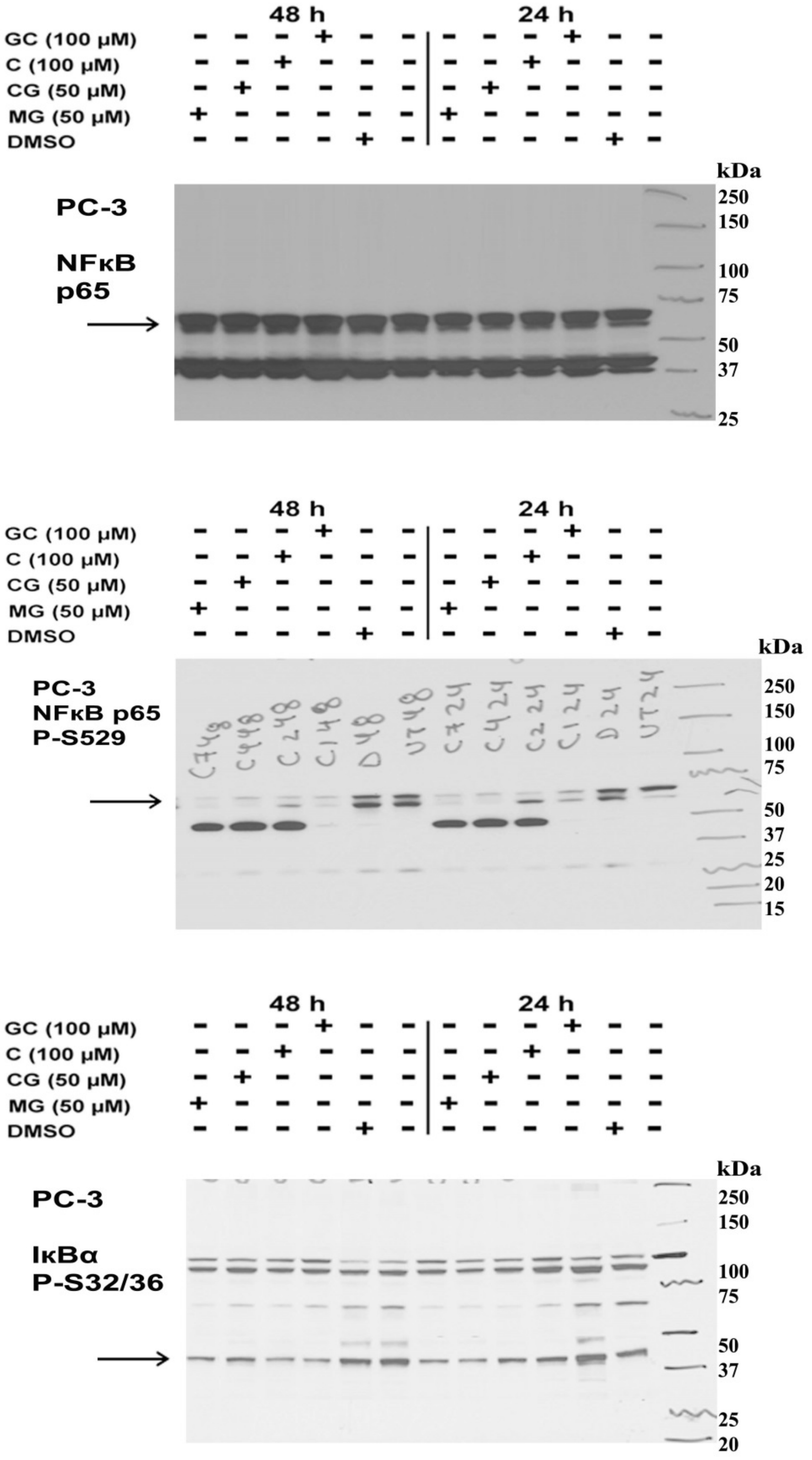
48 h
24 h
GC (100 µM)
C (100 µM)
CG (50 µM)
MG (50 µM)
DMSO
kDa
PC-3
NFκB
p65
250
150
100
75
50
37
25
48 h
24 h
GC (100 µM)
C (100 µM)
CG (50 µM)
MG (50 µM)
DMSO
kDa
PC-3
NFκB p65
P-S529
250
150
100
75
50
37
25
20
15
48 h
24 h
GC (100 µM)
C (100 µM)
CG (50 µM)
MG (50 µM)
DMSO
kDa
PC-3
IκBα
P-S32/36
250
150
100
75
50
37
25
20

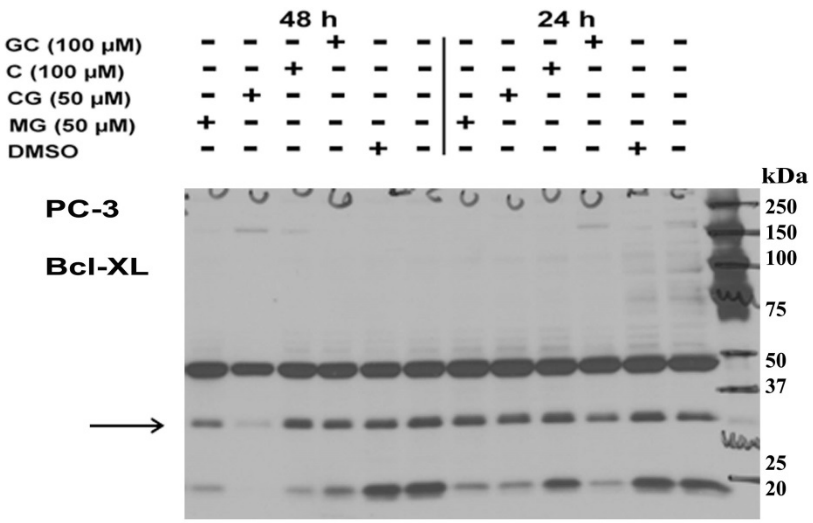
48 h
24 h
GC (100 µM)
C (100 µM)
CG (50 µM)
MG (50 µM)
DMSO
kDa
PC-3
Bcl-XL
250
150
100
75
50
37
25
20

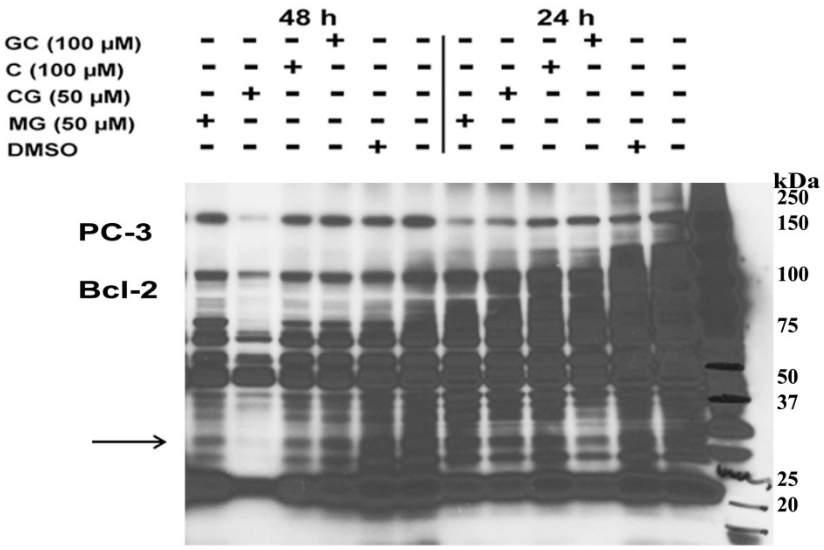
48 h
24 h
GC (100 µM)
C (100 µM)
CG (50 µM)
MG (50 µM)
DMSO
kDa
PC-3
Bcl-2
250
150
100
75
50
37
25
20

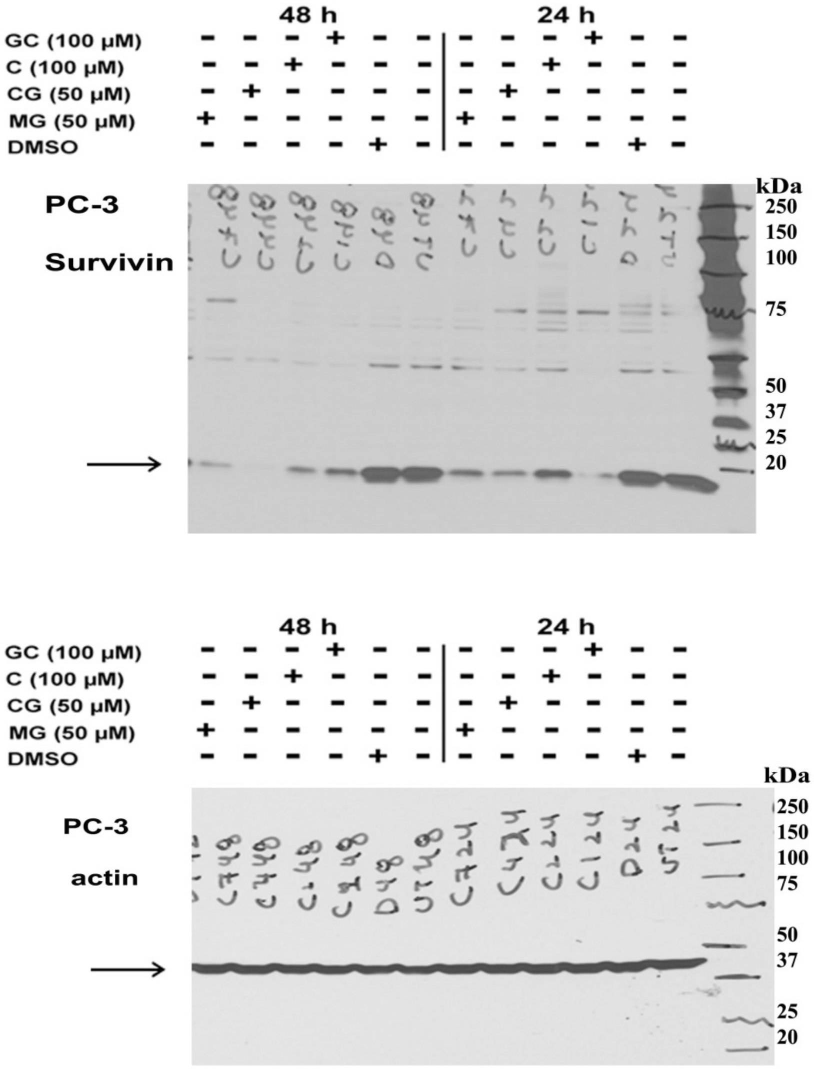

Supplementary Figure 3. Western blot analyses following treatment of PC-3 cells with AHC compounds. Western blot analysis of cellular lysates prepared from PC-3 cells treated with 7-*O*-galloyl catechin (GC), catechin (C), catechin gallate (CG), methyl gallate (MG), or equal dilution of DMSO was performed. Treatments are indicated above the blots. The protein detected is indicated to the left of each blot, and the protein signal detected is indicated by an arrow to the left of each blot. The quantitation of the data is shown in Supplementary Table 2.

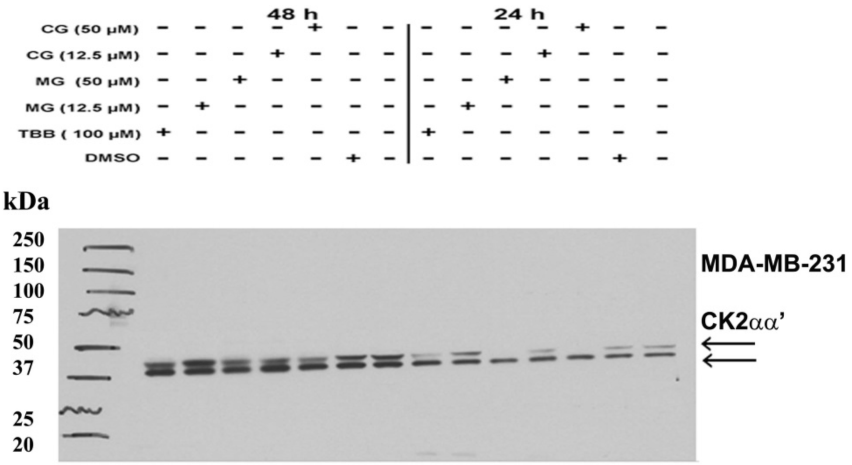
48 h
24 h
CG (50 µM)
CG (12.5 µM)
MG (50 µM)
MG (12.5 µM)
TBB (100 µM)
DMSO
kDa
250
150
100
75
50
37
25
20
MDA-MB-231
CK2αα’

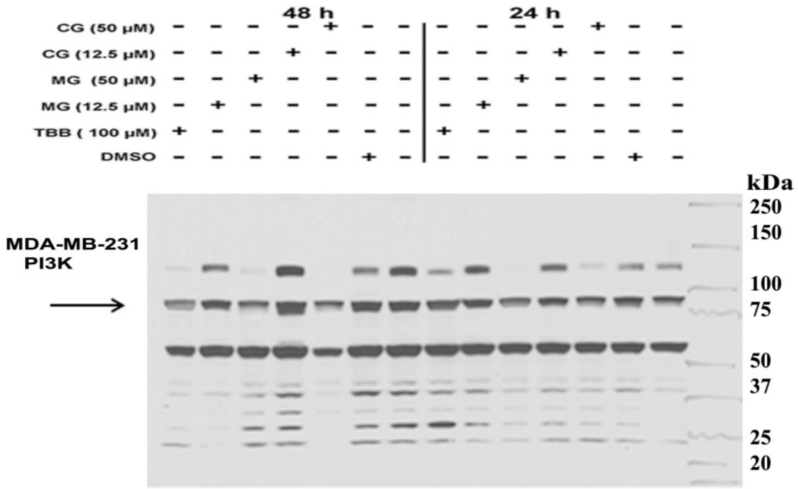
48 h
24 h
CG (50 µM)
CG (12.5 µM)
MG (50 µM)
MG (12.5 µM)
TBB (100 µM)
DMSO
kDa
250
150
100
75
50
37
25
20
MDA-MB-231
PI3K

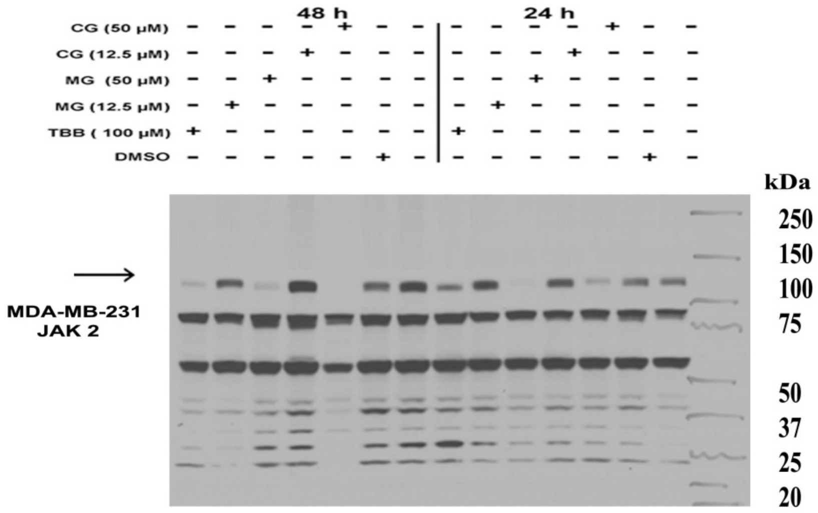
48 h
24 h
CG (50 µM)
CG (12.5 µM)
MG (50 µM)
MG (12.5 µM)
TBB (100 µM)
DMSO
kDa
250
150
100
75
50
37
25
20
MDA-MB-231
JAK 2

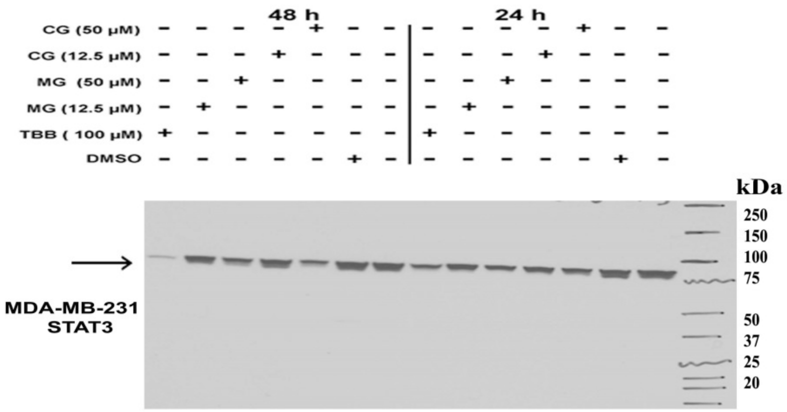
48 h
24 h
CG (50 µM)
CG (12.5 µM)
MG (50 µM)
MG (12.5 µM)
TBB (100 µM)
DMSO
kDa
250
150
100
75
50
37
25
20
MDA-MB-231
STAT3

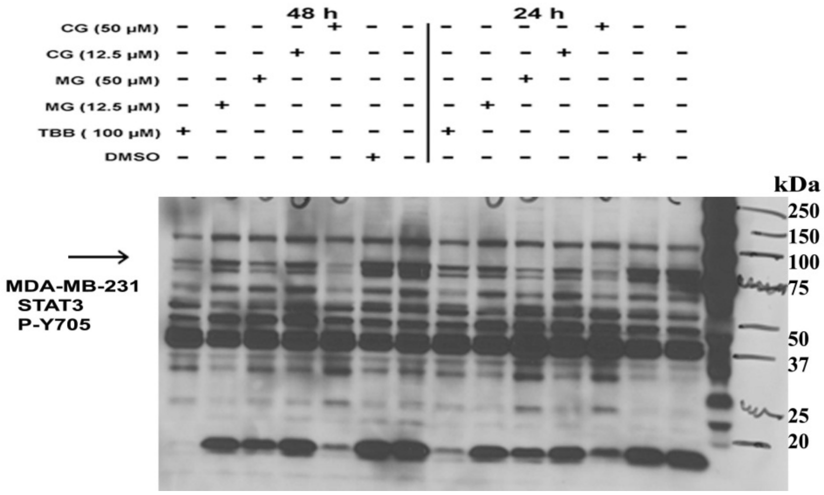
48 h
24 h
CG (50 µM)
CG (12.5 µM)
MG (50 µM)
MG (12.5 µM)
TBB (100 µM)
DMSO
kDa
250
150
100
75
50
37
25
20
MDA-MB-231
STAT3
P-Y705

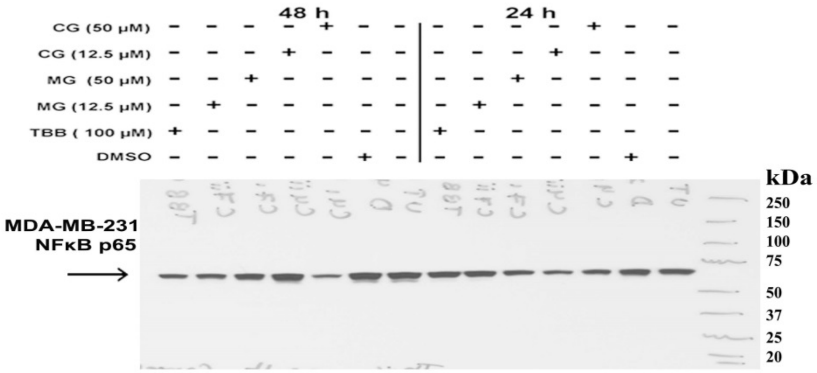
48 h
24 h
CG (50 µM)
CG (12.5 µM)
MG (50 µM)
MG (12.5 µM)
TBB (100 µM)
DMSO
kDa
250
150
100
75
50
37
25
20
MDA-MB-231
NFκB p65

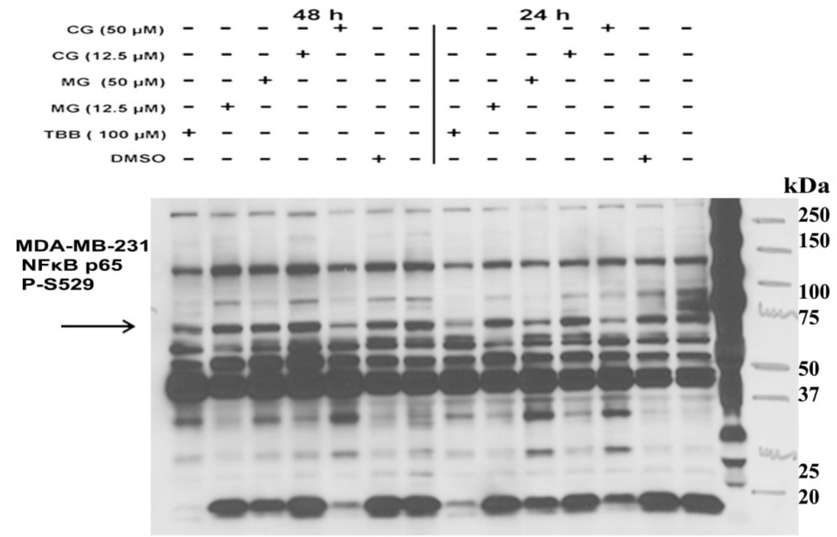
48 h
24 h
CG (50 µM)
CG (12.5 µM)
MG (50 µM)
MG (12.5 µM)
TBB (100 µM)
DMSO
MDA-MB-231
NFκB p65
P-S529
kDa
250
150
100
75
50
37
25
20

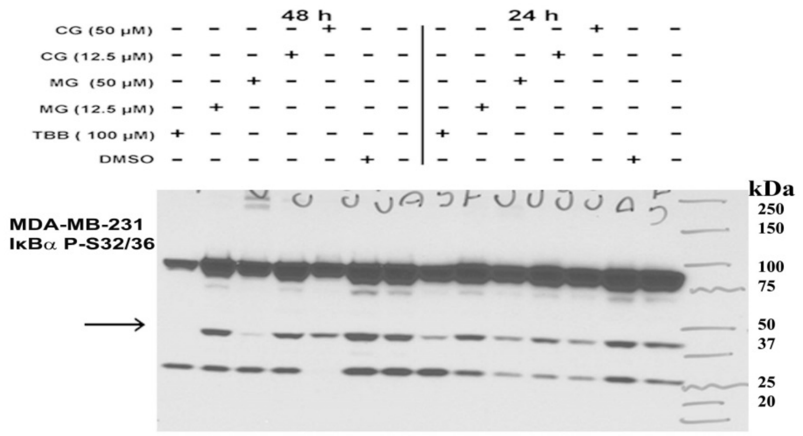
48 h
24 h
CG (50 µM)
CG (12.5 µM)
MG (50 µM)
MG (12.5 µM)
TBB (100 µM)
DMSO
MDA-MB-231
IκBα P-S32/36
kDa
250
150
100
75
50
37
25
20

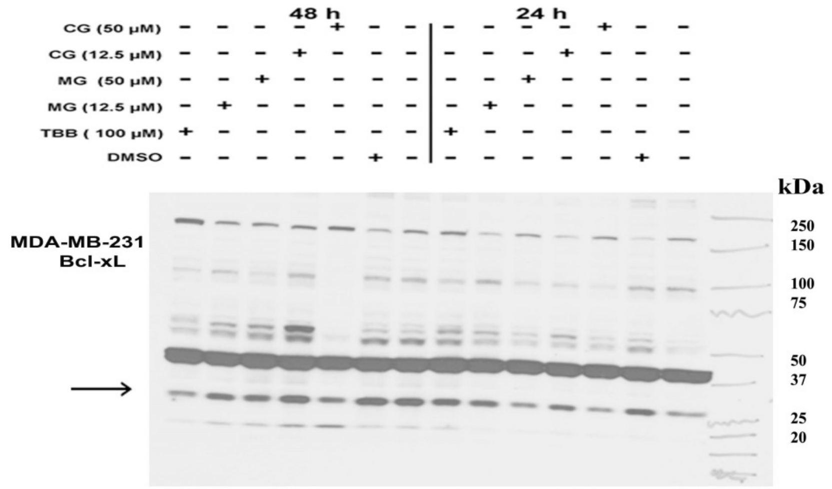
48 h
24 h
CG (50 µM)
CG (12.5 µM)
MG (50 µM)
MG (12.5 µM)
TBB (100 µM)
DMSO
MDA-MB-231
Bcl-xL
kDa
250
150
100
75
50
37
25
20

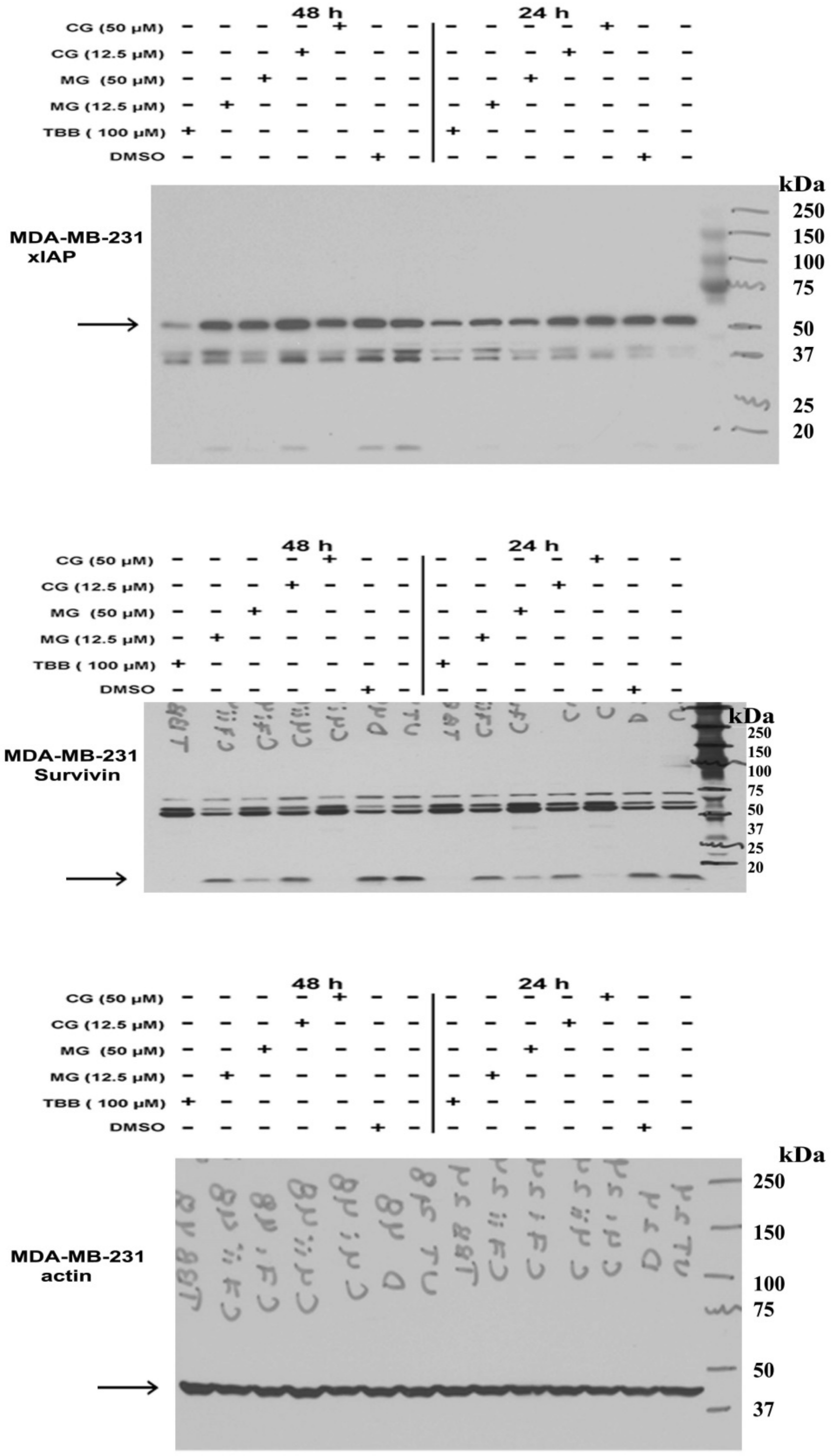

Supplementary Figure 4. Western blot analyses following treatment of MDA-MB-231 cells with AHC compounds. Western blot analysis of cellular lysates prepared from MDA-MB-231 cells treated with 2 concentrations each of catechin gallate (CG) and methyl gallate (MG), or dilution of DMSO representing the highest concentration was performed. Treatments are

indicated above the blots. The protein detected is indicated to the left of each blot, and the protein signal detected is indicated by an arrow to the left of each blot. The quantitation of the data is shown in Supplementary Table 3.

Supplementary Table 1: Cytotoxic effect of *A. hydaspica* active fractions and isolated compounds at 48 h of treatment on MDA-MB-231 and PC-3 cells

Samples	MDA-MB-231	PC-3
**AHE**	39.56±0.51	32.26±0.51
**AHB**	32.65±0.41	35.68±0.45
**AHE fractions/compounds**		
**EF4**	21.36±0.66	25.19±0.91
**EF5**	24.32±0.95	26.36±1.01
**EF6**	22.14±0.87	27.04±0.64
**IF 3 (C4, MG)**	12.30±0.44	10.50±0.85
**IF7 (C2, C)**	-	41.20±0.36
**IF 9**	-	20.51±0.55
**GC**	-	43.50±0.53
**AHB fractions /compounds**		
**BF1**	23.56±0.49	25.56±0.92
**BSF4**	18.59±0.51	19.21±0.55
**CG**	13.30±0.81	10.10±0.61

Each value expressed as mean ± SEM (n=3). IC_{50} values of extract and fractions are presented in µg/ml, whereas IC_{50} values of compounds are in µM. – indicates no effect.

Supplementary Table 2. Effects of various AHC compounds on survival signaling molecules in PC-3 cells.

Signals studied	AHC compounds tested in PC-3 cells (24 h treatment)					AHC compounds tested in PC-3 cells (48 h treatment)				
	GC (100 µM)	C (100 µM)	CG (50 µM)	MG (50 µM)	DMSO *vs* none	GC (100 µM)	C (100 µM)	CG (50 µM)	MG (50 µM)	DMSO *vs* none
CK2α	1.01 ±0.05	0.99 ± 0.04	0.99 ± 0.07	1.01 ± 0.06	1.01 ± 0.074	1.04 ± 0.01	0.96 ± 0.01	0.99 ± 0.02	0.98 ± 0.02	1.02 ± 0.02
CK2α´	0.98 ± 0.03	0.97 ± 0.06	0.97 ± 0.02	0.99 ± 0.05	1.02 ± 0.01	0.94 ± 0.02	0.96 ± 0.04	0.95 ± 0.04	0.97 ± 0.04	1.00 ± 0.01
PI3K	0.99 ± 0.10	1.04 ± 0.08	0.99 ± 0.06	1.03 ± 0.041	1.02 ± 0.01	0.99 ± 0.04	1.00 ± 0.06	0.98 ± 0.07	1.04 ± 0.02	1.05 ± 0.02
JAK2	0.64 ± 0.10[#]	0.51 ± 0.03[#]	0.29 ± 0.01[#]	0.58 ± 0.03[#]	1.05 ± 0.03	0.46 ± 0.08[#]	0.58 ± 0.01[#]	0.18 ± 0.01[#]	0.25 ± 0.04[#]	1.00 ±0.01
Akt	0.895± 0.04	0.89 ± 0.03	0.46 ± 0.09[#]	0.95 ± 0.07	1.02 ± 0.05	0.91 ± 0.04	0.83 ± 0.06*	0.44 ± 0.01[#]	0.52 ± 0.01[#]	1.02 ± 0.05
Akt-P-Ser473	0.48 ± 0.02[#]	0.74 ± 0.02[#]	0.73 ± 0.04[#]	0.46 ± 0.03[#]	1.01 ± 0.05	0.39 ± 0.03[#]	0.71 ± 0.03[#]	0.48 ± 0.02[#]	0.25 ± 0.03[#]	1.01 ± 0.05
Akt-P-Thr308	0.47 ± 0.02[#]	0.54 ± 0.01[#]	0.20 ± 0.01[#]	0.23 ± 0.01[#]	1.00 ± 0.07	0.21 ± 0.01[#]	0.31 ± 0.01[#]	0.20 ± 0.01[#]	0.22 ± 0.01	1.00 ± 0.07
STAT3	0.99 ± 0.04	0.97 ± 0.05	1.01 ± 0.03	1.02 ± 0.04	1.04 ± 0.03	0.97 ± 0.05	0.95 ± 0.09	1.00 ± 0.02	1.00 ± 0.06	1.03 ± 0.04
STAT3 P-Tyr705	0.99 ± 0.41	0.96 ± 0.05	0. 89 ± 0.06	0.94 ± 0.07	1.01 ± 0.05	0.98 ± 0.51	0.91 ± 0.04	0.87 ± 0.05	0.88 ± 0.06	0.99 ± 0.06
ERK 1/2	0.87 ± 0.03	0.91 ± 0.02	0.998 ± 0.03	0.99 ± 0.04	1.01 ± 0.01	0.84 ± 0.02*	0.93 ± 0.04	0.51 ± 0.06[#]	0.93 ± 0.03	1.01 ± 0.01
ERK 1/2 P-Thr202/ P-Tyr204	0.19 ± 0.01[#]	0.80 ± 0.01†	0.63 ± 0.02[#]	0.87 ± 0.02	1.02 ± 0.05	0.19 ± 0.01[#]	0.29 ± 0.02[#]	0.21 ± 0.01[#]	0.10 ± 0.01[#]	1.02 ± 0.05
NFκB p65	0.99 ± 0.05	1.01 ± 0.10	1.14 ± 0.07	1.16 ± 0.31	1.071 ± 0.08	0.99 ± 0.07	1.02 ± 0.11	1.15 ± 0.08	1.20 ± 0.35	1.08 ± 0.09
NFκB p65 P-Ser529	0.22 ± 0.03[#]	0.15 ± 0.03[#]	0.13 ± 0.01[#]	0.12 ± 0.01[#]	1.14 ± 0.11	0.13 ± 0.01[#]	0.11 ± 0.03[#]	0.08 ± 0.01[#]	0.05 ± 0.02[#]	1.01 ± 0.02
IκBα P-Ser32/36	0.29 ± 0.11[#]	0.31 ± 0.12[#]	0.29 ± 0.07[#]	0.31 ± 0.10[#]	1.29 ± 0.20	0.17 ± 0.05[#]	0.20 ± 0.04[#]	0.23 ± 0.02[#]	0.12 ± 0.02[#]	1.02 ± 0.04
Bcl-2	0.93 ± 0.25	0.88 ± 0.02	0.95 ± 0.06	0.92 ± 0.01	0.99 ± 0.04	0.94 ± 0.03	0.81 ± 0.05[#]	0.14 ± 0.04[#]	0.50 ± 0.02[#]	1.00 ± 0.01
Bcl-xL	0.65 ± 0.02[†]	0.84 ± 0.07	0.71 ± 0.05[†]	0.79 ± 0.06*	1.04 ± 0.08	0.72 ± 0.06[#]	0.73 ± 0.07[†]	0.22 ± 0.09[#]	0.48 ± 0.07[#]	1.02 ± 0.07
xIAP	0.96 ± 0.32	0.97 ± 0.14	0.98 ± 0.21	0.97 ± 0.42	1.01 ± 0.20	0.92 ± 0.35	0.94 ± 0.24	0.96 ± 0.51	0.93 ± 0.44	1.04 ± 0.25
Survivin	0.27 ± 0.05[#]	0.45 ± 0.06[#]	0.07 ± 0.03[†]	0.24 ± 0.04[#]	1.00 ± 0.08	0.17 ± 0.05[#]	0.22 ± 0.04[#]	0.01 ± 0.01[#]	0.05 ± 0.04[#]	1.01 ± 0.07

Data are presented as mean ± SEM of at least 3 independent experiments. Data analyzed by two- way ANOVA with Bonferroni post-test. * = $p < 0.05$; **†** = $p < 0.01$; **#** = $p < 0.001$ relative to untreated control cells.

Supplementary Table 3. Effects of various AHC compounds on survival signaling molecules in MDA-MB-231 cells.

Signals studied	AHC compounds tested in MDA-MB-231 cells (24 h treatment)						AHC compounds tested in MDA-MB-231 cells (48 h treatment)					
	CG (50 μM)	CG (12.5 μM)	MG (50 μM)	MG (12.5 μM)	TBB (100 μM)	DMSO *vs* none	CG (50 μM)	CG (12.5 μM)	MG (50 μM)	MG (12.5 μM)	TBB (100 μM)	DMSO
CK2α	0.08 ± 0.04#	0.88 ± 0.03†	0.12 ± 0.04#	0.83 ± 0.15†	0.69 ± 0.02#	1.20 ± 0.08	0.20 ± 0.04#	0.74 ± 0.01*	0.33 ± 0.04#	1.07 ± 0.02	0.68 ± 0.09†	1.03 ± 0.03
CK2α´	0.96 ± 0.03	1.00 ± 0.03	0.93 ± 0.05	1.00 ± 0.10	0.96 ± 0.04	1.01 ± 0.01	0.96 ± 0.03	1.09 ± 0.10	0.97 ± 0.03	1.05 ± 0.04	0.99 ± 0.09	1.04 ± 0.02
PI3K	0.82 ± 0.03	0.98 ± 0.01	0.80 ± 0.08	0.95 ± 0.01	0.94 ± 0.03	0.99 ± 0.01	0.38 ± 0.03#	0.94 ± 0.03	0.33 ± 0.01#	0.90 ± 0.06	0.76 ± 0.06†	0.99 ± 0.02
JAK2	0.25 ± 0.04#	1.17 ± 0.15	0.07 ± 0.02#	1.20 ± 0.10	0.63 ± 0.06†	0.97 ± 0.01	0.04 ± 0.04#	1.04 ± 0.05	0.13 ± 0.05#	0.90 ± 0.04	0.11 ± 0.01#	0.99 ± 0.01
Akt	0.95 ± 0. 11	1.02 ± 0.13	0.96 ± 0.04	1.05 ± 0.31		1.09 ± 0.09	0.92 ± 0.21	1.00 ± 0.12	0.93 ± 0.05	1.00 ± 0.61	-	1.04 ± 0.04
Akt-P-Ser473	1.01 ± 0.22	0.99 ± 0.35	1.03 ± 0.09	1.04 ± 0.15		1.03 ± 0.05	1.00 ± 0.32	0.97 ± 0.55	0.99 ± 0.09	1.00 ± 0.65	-	1.02 ± 0.03
Akt-P-Thr308	1.01 ± 0.22	1.05 ± 0.44	0.98 ± 0.10	1.01 ± 0.21		1.02 ± 0.09	1.00 ± 0.32	1.02 ± 0.54	0.99 ± 0.07	1.02 ± 0.51	-	1.05 ± 0.08
STAT3	0.50 ± 0.04#	0.64 ± 0.03#	0.54 ± 0.07#	0.62 ± 0.04#	0.48 ± 0.02#	1.02 ± 0.08	0.20 ± 0.03#	0.75 ± 0.03†	0.42 ± 0.08#	0.76 ± 0.05†	0.12 ± 0.03#	1.00 ± 0.04
STAT3 P-Tyr705	0.20 ± 0.01#	0.34 ± 0.05#	0.15 ± 0.03#	0.35 ± 0.05#	0.26 ± 0.04#	1.04 ± 0.12	0.15 ± 0.02#	0.44 ± 0.04#	0.15 ± 0.03#	0.46 ± 0.05#	0.24 ± 0.03#	1.01 ± 0.06
ERK 1/2	1.02 ± 0.34	0.97 ± 0.14	0.96 ± 0.19	0.99 ± 0.06	0.90 ± 0.01	1.04 ± 0.03	1.00 ± 0.54	0.92 ± 0.34	0.92 ± 0.09	0.95 ± 0.05	0.91 ± 0.05	1.02 ± 0.04
ERK 1/2 P-Thr202/ P-Tyr204	0.95 ± 0.21	0.97 ± 0.06	0.99 ± 0.03	1.00 ± 0.06	0.61 ± 0.08#	1.01 ± 0.05	0.94 ± 0.30	0.95 ± 0.06	0.97 ± 0.07	1.00 ± 0.06	0.75 ± 0.04†	0.99 ± 0.03
NFκB p65	0.60 ± 0.01	0.95 ± 0.03	0.71 ± 0.04	1.01 ± 0.03	0.21 ± 0.04	1.07 ± 0.01	0.21 ± 0.04#	0.62 ± 0.32	0.59 ± 0.04#	0.74 ± 0.02	0.65 ± 0.13	1.07 ± 0.01
NFκB p65 P-Ser529	0.49 ± 0.06#	1.03 ± 0.02	0.56 ± 0.12#	0.87 ± 0.10	0.60 ± 0.15#	1.10 ± 0.01	0.43 ± 0.01#	0.89 ± 0.04	0.55 ± 0.03#	0.84 ± 0.08	0.58 ± 0.09#	1.10 ± 0.05
IκBα P-Ser32/36	0.07 ± 0.03#	0.52 ± 0.01#	0.08 ± 0.02#	0.96 ± 0.03	0.09 ± 0.02#	1.12 ± 0.01	0.07 ± 0.02#	0.49 ± 0.04	0.07 ± 0.04#	0.71 ± 0.04#	0.01 ± 0.05#	1.21 ± 0.06
Bcl-2	0.98 ± 0.14	1.02 ± 0.09	0.92 ± 0.52	0.96 ± 0.11	0.97 ± 0.04	1.02 ± 0.05	0.99 ± 0.21	1.00 ± 0.09	0.90 ± 0.51	0.93 ± 0.11	0.94 ± 0.03	1.01 ± 0.04
Bcl-xL	0.31 ± 0.09#	0.50 ± 0.05	0.39 ± 0.07#	0.51 ± 0.04#	0.83 ± 0.03	1.19 ± 0.11	0.15 ± 0.06#	0.79 ± 0.01	0.11 ± 0.05#	0.16 ± 0.04#	0.09 ± 0.02#	1.17 ± 0.09
xIAP	0.90 ± 0.04	0.95 ± 0.04	0.81 ± 0.05	0.89 ± 0.06	0.21 ± 0.01#	1.13 ± 0.05	0.82 ± 0.05*	0.95 ± 0.04	0.56 ± 0.02#	0.81 ± 0.06*	0.21 ± 0.01#	1.13 ± 0.05
Survivin	0.09 ± 0.02#	0.71 ± 0.01#	0.41 ± 0.03#	0.71 ± 0.04#	0.06 ± 0.03#	1.09 ± 0.01	0.06 ± 0.04#	0.69 ± 0.01#	0.39 ± 0.03#	0.67 ± 0.03#	0.01 ± 0.01#	1.03 ± 0.01

Data are presented as mean ± SEM of at least 3 independent experiments. Data analyzed by two- way ANOVA with Bonferroni post-test. * = $p < 0.05$; † = $p < 0.01$; # = $p < 0.001$ relative to untreated control cells.
